# Transcriptome Profiling of Bovine Macrophages Infected by *Mycobacterium avium* spp. *paratuberculosis* Depicts Foam Cell and Innate Immune Tolerance Phenotypes

**DOI:** 10.3389/fimmu.2019.02874

**Published:** 2020-01-08

**Authors:** Olivier Ariel, Daniel Gendron, Pier-Luc Dudemaine, Nicolas Gévry, Eveline M. Ibeagha-Awemu, Nathalie Bissonnette

**Affiliations:** ^1^Sherbrooke Research and Development Center, Agriculture and Agri-Food Canada, Sherbrooke, QC, Canada; ^2^Department of Biology, Université de Sherbrooke, Sherbrooke, QC, Canada; ^3^Department of Biochemistry, Université de Sherbrooke, Sherbrooke, QC, Canada

**Keywords:** *Mycobacterium avium* spp. *paratuberculosis*, bovine, paratuberculosis, macrophage, innate immunity, immune tolerance, RNA-seq, Johne's disease

## Abstract

*Mycobacterium avium* spp. *paratuberculosis* (MAP) is the causative agent of Johne's disease (JD), also known as paratuberculosis, in ruminants. The mechanisms of JD pathogenesis are not fully understood, but it is known that MAP subverts the host immune system by using macrophages as its primary reservoir. MAP infection in macrophages is often studied in healthy cows or experimentally infected calves, but reports on macrophages from naturally infected cows are lacking. In our study, primary monocyte-derived macrophages (MDMs) from cows diagnosed as positive (+) or negative (–) for JD were challenged *in vitro* with live MAP. Analysis using next-generation RNA sequencing revealed that macrophages from JD(+) cows did not present a definite pattern of response to MAP infection. Interestingly, a considerable number of genes, up to 1436, were differentially expressed in JD(–) macrophages. The signatures of the infection time course of 1, 4, 8, and 24 h revealed differential expression of *ARG2, COL1A1, CCL2, CSF3, IL1A, IL6, IL10, PTGS2, PTX3, SOCS3, TNF*, and *TNFAIP6* among other genes, with major effects on host signaling pathways. While several immune pathways were affected by MAP, other pathways related to hepatic fibrosis/hepatic stellate cell activation, lipid homeostasis, such as LXR/RXR (liver X receptor/retinoid X receptor) activation pathways, and autoimmune diseases (rheumatoid arthritis or atherosclerosis) also responded to the presence of live MAP. Comparison of the profiles of the unchallenged MDMs from JD(+) vs. JD(–) cows showed that 868 genes were differentially expressed, suggesting that these genes were already affected before monocytes differentiated into macrophages. The downregulated genes predominantly modified the general cell metabolism by downregulating amino acid synthesis and affecting cholesterol biosynthesis and other energy production pathways while introducing a pro-fibrotic pattern associated with foam cells. The upregulated genes indicated that lipid homeostasis was already supporting fat storage in uninfected JD(+) MDMs. For JD(+) MDMs, differential gene expression expounds long-term mechanisms established during disease progression of paratuberculosis. Therefore, MAP could further promote disease persistence by influencing long-term macrophage behavior by using both tolerance and fat-storage states. This report contributes to a better understanding of MAP's controls over the immune cell response and mechanisms of MAP survival.

## Introduction

*Mycobacterium avium* subsp. *paratuberculosis* (MAP) is an obligate intracellular pathogen causing paratuberculosis, an incurable disease also known as Johne's disease (JD). This chronic inflammatory disease induces a granulomatous enteritis (1). The MAP bacterium affects ruminants, both domestic and wild, around the world, affects livestock productivity, and jeopardizes animal health (2). Additionally, there is a long-standing debate on the precise role played by MAP in Crohn's disease pathogenesis in humans. Because MAP introduces some symptoms in ruminants similar to those of Crohn's disease, this pathogen will remain an incriminating zoonotic factor until conclusive proof is established (3–5).

Different infection models have been used to study disease progression. The host–MAP cross-talk turns into a duel that lasts for years with unpredictable disease progression. When infecting macrophages, MAP elicits an initial strong T helper type 1 (Th1)-mediated response that is dominated by interferon gamma (IFN-γ)–secreting T cells (6, 7). The Th1 response is normally effective at controlling intracellular infections such as those caused by non-pathogenic mycobacteria, but the host is unable to clear MAP infection because MAP has developed strategies to survive within infected macrophages (6). As JD progresses, Th1 response subsides and a non-protective T helper type 2 (Th2) response becomes prominent (7, 8). This Th1-to-Th2 switch dogma was stated because the dominating IFN-γ response was barely detected in the later stages of the disease (9). This model has been challenged from time to time by subsequent studies, since the conversion to a Th2 humoral immune response is not the most accurate description of the progression of the disease (10–12). As JD progresses, T cell hyporesponsiveness is observed (11), which is also documented as T cell exhaustion (13, 14) and/or anergy (12). Alternatively, it was suggested that another subtype of T cells, the regulatory T cells (Treg), play a regulatory role in JD (13–15). Of particular relevance is the interplay between Treg and T helper type 17 (Th17) cells producing interleukin-17 (IL-17) (16). Higher level of IL-17 has been detected in naturally MAP-infected animals (11). JD also appears to affect other host processes, such as the ability of macrophages to become activated by IFN-γ and to produce nitric oxide (17). For its long-term survival, MAP modifies first its microenvironment which might shape the local immune response.

It is known that MAP uses macrophages as its primary reservoir. Therefore, macrophages have been used as a model to study the pro- or anti-inflammatory shift influencing the effector functions of the immune cells following MAP exposure. To investigate the effect of MAP on gene expression, human monocytic cell lines (18, 19), bovine monocytes, or monocyte-derived macrophages (MDMs) from cows (20–23) have been used. The two well-studied polarized macrophage subsets M1 and M2, which are conceptually analogous with Th1 and Th2 immune responses (24), have been described for bovine. When experimentally infected with MAP, a heterogeneous monocyte/macrophage population with a mixed phenotype has been observed in calves (20). In our study, primary MDMs from naturally MAP-infected and JD-free cows were used in order to study *in vitro* their response to living MAP.

There is a wealth of evidence suggesting the role of pathogen- and host-associated lipids in the survival and persistence of pathogenic MAP (25). Interestingly, phagosome maturation and lysosomal fusion are affected only by living MAP, since dead bacilli cannot prevent these processes (18, 26) similar to what is observed for *Mycobacterium tuberculosis* (27). For invasion, MAP interacts with cell receptors such as toll-like receptors (TLRs), DC-SIGN (dendritic cell-specific intercellular adhesion molecule-3-grabbing non-integrin), CD14, CR3 (complement receptor), and mannose receptors (28, 29). The TLR signaling pathways are one of the survival strategies that MAP uses to escape the host's defense mechanisms. For instance, following TLR2 recognition, MAP activates MAPK-p38, which results in an inhibition of phagosome maturation, independent to the upregulation of interleukin-10 (IL-10) (30, 31). Other studies reported the role of metal transporters such as SLC11A1, which pumps out divalent cation from the phagosome as a host strategy to restrict pathogen growth by iron deprivation (32). However, MAP deploys different strategies to create its protected microenvironment. Notably, by preventing the maturation and acidification of the phagosome, MAP facilitates its persistence (18), as observed also in *M. tuberculosis* infection (33, 34). The macrophage–MAP cross-talk is most likely the main sustained activity that the host has to deal with during infection.

The purpose of our study was to better understand the macrophage–MAP cross-talk during the subclinical and MAP shedding stage of the disease. Primary MDMs from naturally infected cows were investigated using next-generation RNA sequencing (RNA-seq). Using a clinical strain of MAP, the response of the macrophages to an *in vitro* MAP infection was analyzed to characterize the biological processes and biological pathways enriched by the differentially expressed (DE) genes that MAP induces to establish a persistent infection. This is the first study that contributes to a better understanding of the mechanisms used by MAP to control blood-circulating immune cells during the subclinical stage of the disease.

## Methodology

### Ethical Approval, Herd Selection, and Consent to Participate

All animal procedures were carried out according to the Canadian Council on Animal Care guidelines for institutional animal use (ethical approval protocols 424 and 431). The source and target populations were, respectively, the dairy cow herds enrolled in the voluntary program for the prevention and control of paratuberculosis in Quebec and the herds in which a JD clinical case had been reported during the previous year. Enrollment of JD-positive herds was confirmed using environmental samples and using pools of dry cow and sick cow fecal samples. Similar criteria are described elsewhere (35). The owners of all animals used in this study signed a collaborative agreement allowing the use of their animals.

### Animal Selection and JD Diagnosis

Sixteen commercial dairy farms (tie and free stall) in the province of Québec, Canada, were selected for this study. Blood was sampled from animals and tested for the presence of MAP antibodies using the Pourquier ELISA assay (IDEXX Laboratories, Markham, Ontario, Canada) according to the manufacturer's instructions. Feces was also sampled from animals and fecal excretion of MAP was confirmed using the mycobacterial culture method by the Laboratoire d'épidémiosurveillance animale du Québec (Saint-Hyacinthe, Québec, Canada), as previously described (35). The cows were negative [JD(–)] if both tests (serum ELISA and fecal culture) were negative (–/–; *n* = 6) and the second group of cows were positive [JD(+)] if both tests were positive (+/+; *n* = 6). These animals (*n* = 12) were selected for the RNA-seq analysis. The 6 JD(–) cows were from herds with no past history of JD while the 6 JD(+) cows and additional 37 cows [18 JD(–) and 19 JD(+)] were selected from JD(+) herds. These additional cows (*n* = 37) were used for RT-qPCR validation of the RNA-seq data. These animals were tested every 6 months during a 3–5 year period as described (36). Their sera were analyzed using the Map detection ELISA kit (IDEXX Laboratories) and fecal excretion of MAP DNA was confirmed by PCR. Confirmation of both tests during the longitudinal study was required to classify the cows as negative (–/–; *n* = 18) or positive (+/+; *n* = 19) for the RT-qPCR validation analysis. At the time blood samples were taken for *in vitro* experiments, JD(–) cows from the validation group were specifically selected to be in at least their second lactation to increase the likelihood of selecting truly negative cows. Meanwhile, these JD(–) cows from the JD(–) herds were continuously monitored thereafter, every 6 months until they were culled aged >7 years old, with no history of intermittent shedding and with a constant basal blood ELISA scores (<0.1 S/P). Feces from JD(–) cows were analyzed by cultural and qPCR methods to check for the absence of MAP. The mean age of JD(–) cows was 6.4 ± 1.2 years and was 5.1 ± 1.4 years for the JD(+) cows at the time *in vitro* experiments were conducted.

### Monocyte Isolation and MDM Differentiation

Peripheral blood mononuclear cells (PBMCs) were isolated from 700 ml of blood drawn from the jugular vein using two commercial transfusion bags containing citrate–phosphate–dextrose–adenine anticoagulant (Animal Blood Resources International, Dixon, CA, USA). The methods used to isolate PBMCs and monocytes by adherence and to culture the MDMs have previously been described (11, 37). The identity and purity of CD14+ monocytes were confirmed by flow cytometry using Pacific Blue anti-human CD14 (clone M5E2) antibody as described previously (11). The purity of CD14+ cells on day 2 was estimated at ≥90%. For differentiation of monocytes into macrophages, cultures were maintained for 7–10 days in a humid atmosphere at 39°C with 5% CO_2_. The culture medium was refreshed every 2 to 3 days. Macrophage purity was validated by flow cytometry using anti-CD68 antibody, targeting a macrophage specific lysosomal-associated membrane protein (38). The cells were fixed and permeabilized using Cytofix/cytoperm kit (BD Biosciences, Mississauga, Ontario, Canada). The primary antibody was a mouse monoclonal anti-human CD68 (M071801-5; Agilent) and the secondary antibody was a rat anti-Mouse PE labeled IgG1 (P-21129; ThermoFisher, Waltham, MA, USA). The flow cytometry analysis was performed on the 3-laser FACSCanto II flow cytometer (BD Biosciences). After 7–10 days, differentiation was estimated at 90 ± 3%. Once the cell displayed macrophage morphology, the culture medium was replaced with a complete media without antibiotics/antimycotics. The macrophages were then incubated overnight under the same conditions for the MAP infection experiment on the following day.

### Bacteria Preparation and *in vitro* MAP Infection of Primary Macrophages

A clinical field strain of MAP (MAP 39382; type Cattle) was cultured (passages 6–10) using Middlebrook 7H9 liquid media supplemented with mycobactin J (Allied Monitor, Fayette, MO, USA) at a final concentration of 2 μg/L, 100 ml/L of oleic albumin dextrose catalase (OADC) growth supplement (BD Biosciences), and 2% (v/v) glycerol (Bioshop, Burlington, Ontario, Canada). The absorbance (A_600nm_) of the bacterial suspension was measured weekly to assess bacterial growth. After 10 weeks, bacteria were harvested, washed with sterile phosphate buffer saline (PBS) (pH 7.4), and then resuspended in PBS. The suspension was passed through a 26-gauge needle to disperse the clumps and lay for 30 min to decant clumps, and MAP concentration was evaluated by absorbance using the formula 0.3 at OD_600_ = 10^9^ CFU/ml (22), validated by microscopic visual examination for enumeration of MAP as described (39), and also by cultural MGIT and colony-forming unit methods as described previously (40). Cell culture and infections were performed at 39°C, the body temperature at which MAP infectivity is enhanced in bovine (41). The experimental design of the MDM infection is shown in Supplementary Figure 1. Infections were performed for incubation periods of 1, 4, 8, and 24 h at a multiplicity of infection (MOI) of 10 bacteria for each macrophage. Uninfected control flasks were also harvested at 4 and 24 h. To stop the infection, cells were washed twice with PBS and harvested by adding 1.8 ml of RLT buffer (Qiagen, Toronto, Ontario, Canada) to each culture flask. Lysed cells were stored at −80°C until required for RNA extraction.

### Detection of MAP in Macrophages

The MDMs were seeded at a density of 1 million cells on a glass coverslip and cultured in a six-well plate under the same conditions as described above. After MAP infection, the MDMs were washed with RPMI and then fixed using ice-cold 100% ethanol. The coverslips were then stained with an acid-fast staining using the commercial BD Auramine M kit protocol (BD Biosciences) and a DAPI 300 nM solution (Roche Diagnostics, Indianapolis, IN, USA) for 30 min. Coverslips were mounted on a microscope slide using Fluoroshield (Sigma-Aldrich, St. Louis, MO, USA) and examined using the Zeiss Axio Observer Z1 (Zeiss Canada, Toronto, Ontario, Canada). Analyses were performed using the ZEN 2 (blue edition) v2.0.0.0 software at the imaging platform of the Department of Biology of the Université de Sherbrooke (Quebec, Canada). All images were taken with the 63× objective and consisted of a merged image of the eGFP, DAPI, and differential interference contrast (DIC) channels. To confirm the absence of MAP in control samples from both JD(–) and JD(+) cows, DNA was extracted from adherent monocytes, MDM, and PBMC using ZR Fecal DNA MiniPrep kit (Zymo Research Corp., Irvine, CA, USA) and qPCR was performed using the VETMAX Gold MAP Detection Kit (Life Technology Inc., Burlington, Ontario, Canada) as described previously (36).

### Lipid Accumulation Assay

The MDMs were seeded at a concentration of 5 × 10^5^ per well in 24-well plates (Corning, New York, NY, USA). Macrophages were infected with MAP at a MOI of 10 as described in section Bacteria preparation and *in vitro* MAP infection of primary macrophages for 24 h, washed using PBS, and cultured in complete media for an additional 5 days, as described previously (33). The lipids were stained using Oil Red O (ORO) as described by Xu et al. (42). The samples from one JD(+) cow and one JD(–) cow including all experimental infection conditions were simultaneously treated to eliminate any bias. The experiment was repeated with four additional cows (JD +/–) and included technical replicates (at least three MAP infected and three CTL wells). Lipid ORO staining was observed using the light microscopy channel of the Evos FL Auto microscope (Thermo Fisher). Photography and image processing were performed using the included EVOS FL Auto Software Revision 1.6 (Thermo Fisher).

### RNA Extraction, Library Preparation, and Sequencing

For RNA-seq analysis (MDM from 12 cows), RNA was extracted using RNeasy kit (Qiagen) with the on-column DNase treatment according to the manufacturer's recommendations. The RNA from the additional 37 animals used for qPCR validation were extracted using the miRNeasy kit (Qiagen), according to the manufacturer's recommendations, which allows for the extraction of both total RNA for qPCR validation and small RNA for future analysis. The RNeasy classic protocol does not retain small RNA in the final fraction. The RNA yield of the samples was quantified using a NanoDrop spectrophotometer (Thermo Fisher). The quality was assessed using the Bioanalyzer RNA 6000 kit (Agilent Technologies, Santa Clara, CA, USA).

Following rRNA removal using the Ribo-Zero Gold kit (Illumina, Victoria, British Columbia, Canada), 72 cDNA libraries (12 animals × 6 samples, including infection and control time points) were generated using 250 ng of total RNA/sample and the Illumina TruSeq Stranded Total mRNA Sample Preparation kit (Illumina). The length of the fragment was assessed using the Agilent High Sensitivity DNA Chip (Agilent Technologies) on a 2100 Bioanalyzer (Agilent Technologies), and the concentration was determined using a NanoDrop spectrophotometer. Libraries were multiplexed in equal ratios (three libraries per lane) and sequenced 100-bp paired-end reads on a HiSeq 2000 instrument (Illumina) by McGill University and Génome Québec Innovation Center (MUGQIC) (http://gqinnovationcenter.com/). RNA-seq data have been deposited in the NCBI Gene Expression Omnibus (GEO) database under the accession number GSE98363.

### RNA-Seq Read Preparation and Alignment

A minimum of 60 million paired reads were obtained for each sample. The bioinformatic analysis was performed using the pipeline RNA-seq pipeline v.1.2 developed by the MUGQIC team. Briefly, trimming and clipping were done using Trimmomatic software v0.32. Trimming was performed by removing extremities that had a phred quality score below 20. Clean reads with a minimum length of 32 nucleotides were aligned using TopHat software v2.0.9 to the Btau 4.6.1 reference genome, allowing for two mismatches per read. The quality of the alignment was verified by checking for high levels of duplicated reads and low transcript coverage with the RNA-SeQC package v1.1.7. Further analysis focused on uniquely mapped reads to calculate the gene count. These unique reads were used to calculate the fragments per kilobase per million mapped reads (FPKM) of each gene for downstream differential gene expression and system biology analyses. Transcripts covered by fewer than five read counts were filtered out to remove traces of trivial gene expression.

### RNA-Seq Gene Expression Evaluation

The FPKM calculated by Cufflinks software v2.1.1 was used to determine the transcript expression rate. Correlation analyses between samples were performed on the FPKM gene expression values to discriminate our samples and identify outsider samples using the *cor* function from the *stats* package of R software v3.1.2. All samples with a correlation value of <50% with the other biological replicates for their corresponding time point and JD status group were eliminated from subsequent analyses. To examine the global sample distribution, principal component analysis (PCA) plots of the FPKM values of each sample and time point were drawn using the *stats R* package. The PCA graphs were produced for the sample of each JD group separately and also using total samples from both groups. All R analyses and graphs were processed in RStudio v0.99.467. Cuffdiff v3.10 software was used to identify genes DE between groups. Two types of comparisons were performed using Cuffdiff. The effect of infection in each JD status group was evaluated by comparing the 12 controls (merged CTL 4 h and CTL 24 h replicates) to each infection time point (1 h, 4 h, 8 h, and 24 h). Second, the effect of the JD status was assessed by comparing CTLs macrophages from the JD(–) cows to the CTLs from JD(+) cows.

### Pathway Enrichment Analysis

Pathway enrichment analysis was performed using the Ingenuity Pathway Analysis (IPA) software (summer release, June 2016, Qiagen). This was used to depict the biological influence of the DE genes identified in all the comparisons carried out in the RNA-seq analysis. Significantly DE genes were analyzed through a functional clustering analysis to identify biological functions affected by those changes using Fisher's exact test. The IPA software also calculated a pathway activation and inhibition score (*z* score) using the DE genes fold-change values in addition to the number of DE gene enrichment of the pathway.

### RT-qPCR Validation Analysis

The RNA from MDM (four time points and controls) from the 49 animals was reversed transcribed using 1 μg of total RNA with SuperScript II reverse transcriptase (Life Technologies Inc.). The primers were designed using Primer Express software (Thermo Fisher) (Supplementary Table 1). The relative quantification of gene expression was determined using standard curves. Normalization was performed using the geometric mean of *PPIA* and *UXT*, which were identified as being among the five most stable housekeeping genes of primary bovine macrophages (11). Triplicate qPCR reactions were performed with the Fast SYBR Green Master Mix buffer (Thermo Fisher) in a final volume of 10 μl. Reactions were read on a 7500 Real-Time PCR System (Thermo Fisher). The qPCR validation focused on the control, 4-h, and 8-h time points because they had the greatest variation and biological meaning.

### Statistical Analysis

For RNA-seq data analysis, DE genes were defined as significant with a *p* < 0.05 after FDR correction (*q* value) using the approach of Benjamini and Hochberg. Genes having a fold-change >2 were considered in order to take into account the biological importance of the DE difference. Uninfected 4 and 24 h control samples were used as biological replicates to increase the statistical power. The statistical analysis of qPCR results was performed using SAS/STAT software (SAS Canada, Toronto, Ontario, Canada). Since the relative expressions returned non-normal distribution for all studied genes, a non-parametric one criterion variance analysis was done using the Kruskal–Wallis test. The differential gene expression was considered significant for the qPCR assay when the result of the Kruskal–Wallis test was below 0.05.

## Results

### Infection Efficiency Was Similar for JD(–) and JD(+) Macrophages

**Figure 1:**
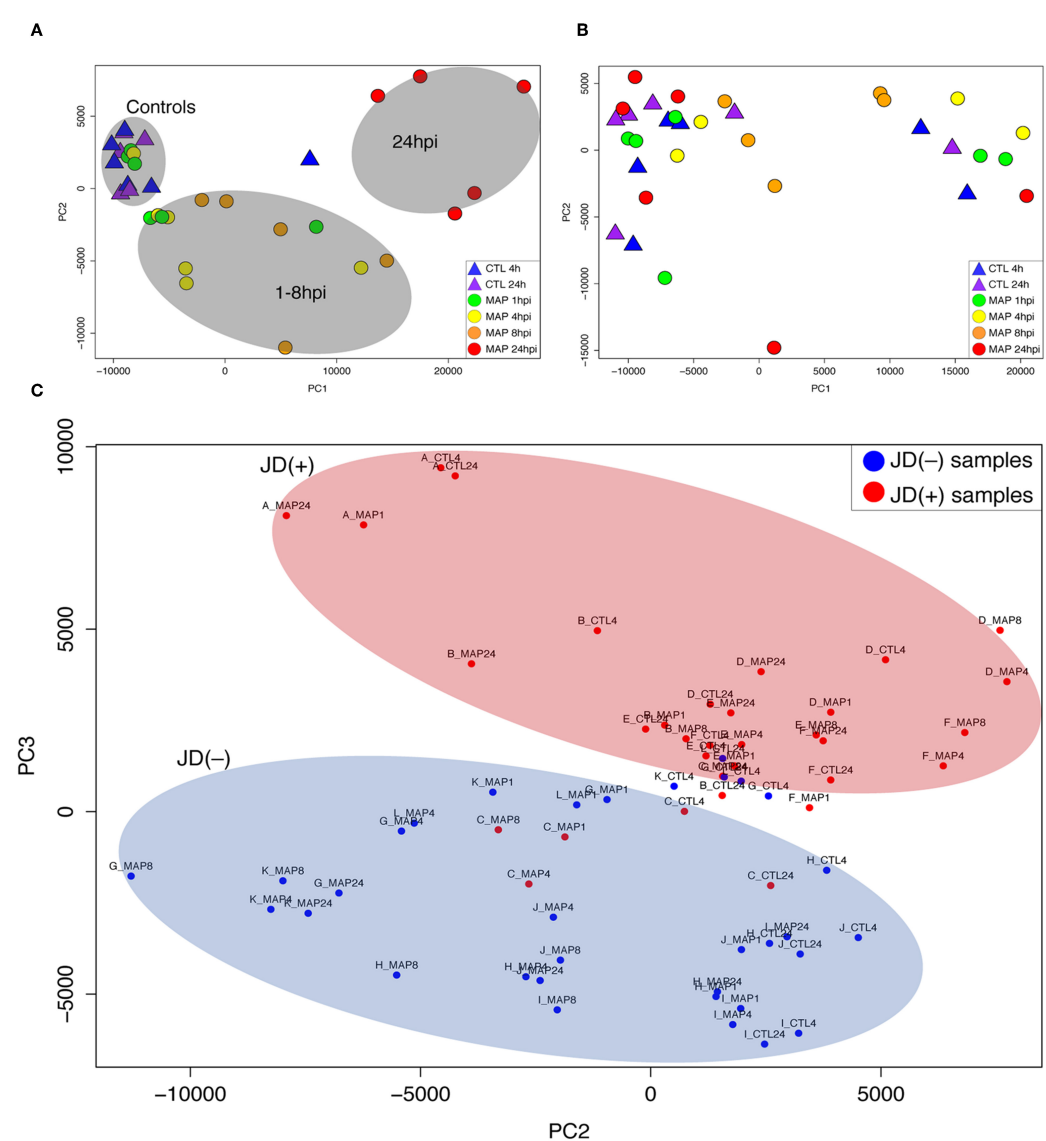
Principal component analysis (PCA) of RNA-seq expression values from macrophages of Johne's disease (JD) negative [JD(–)] cows **(A)**, JD positive [JD(+)] cows **(B)**, and analysis of macrophages from both JD status groups **(C)**. **(A,B)** report the first (PC1) and second (PC2) components, while (**C**) represents the second (PC2) and third (PC3) components. JD(–) cows are designated by letters A–F and JD(+) cows are designated by letters G–L. Each graph shows controls (CTL 4 h and 24 h) and MAP infection (MAP 1, 4, 8, and 24 h) time points.

Primary MDMs from both JD groups were challenged *in vitro* with live MAP. Different time points were analyzed, including 1, 4, 8, and 24 h post infection (hpi) in addition to unchallenged control (CTL) as shown in Figure 1. In the CTL samples of both JD groups, no MAP was detected by either qPCR or fluorescence microscopy (Supplementary Figure 2). Moreover, no MAP was detected in the genomic extracts from the PBMCs and the isolated monocytes before differentiation (data not shown). MAP uptake by the macrophages was similar in both JD status groups (Supplementary Figure 2, Supplementary Table 2). The infection of the MDMs was found to be widespread, with more than 90% of cells being infected at 8 and 24 hpi, while freshly differentiated macrophages from either JD(–) or JD(+) cows were free of MAP (Supplementary Figure 2). There was no significant difference between the two JD groups. Taken together, these results indicate that freshly isolated monocytes were not infected prior to MDM culture and their capacity to phagocytose MAP was similar for both JD(–) and JD(+) macrophage populations.

### Depth RNA-Seq for Detecting Genes Across the Infection Times

The 72 RNA-seq libraries generated an average of 109 million paired-end reads per library, with an average read length of 88 bases after adapters and poor-quality sequences had been removed. After trimming and alignment to the bovine reference genome, 62.8% of the reads mapped to unique locations, whereas others mapped to multiple locations, ribosomal sequences, or mitochondrial sequences. Among the 72 libraries, a mean of 9691 assembled transcripts were identified, and 96.8% were annotated (9383 genes per library). No significant difference (*p* = 0.4207) in the number of detected genes was observed between the JD status groups (Supplementary Table 3). Sequence coverage was adequate, as highly expressed genes in infected samples did not mask less abundant genes. In addition, the number of genes detected was similar across the different time points and among the 72 samples. A large proportion of mapped reads (57.76%) were exonic sequences, and the remaining reads were intronic (8.96%) and intergenic (33.16%) sequences. Therefore, the expression spectrum at different infection time points of all the 9691 assembled transcripts was covered, allowing detection of both highly expressed and low-abundant transcripts.

### MAP Induced a Major Effect on the Transcriptome of JD(–) Macrophages but Not of JD(+) Macrophages

PCA was used to capture similarities and variations within this large multivariate dataset of 72 RNA-seq samples. These representations helped identify correlations between the different transcriptomic profiles in response to MAP infection. As shown in Figures 1A,B, the JD(–) and JD(+) samples showed distinct patterns. With the exception of one outsider sample (one uninfected CTL at 4 h) that was removed from the downstream analysis, all the CTL samples from JD(–) cows were grouped together (Figure 1A). The distribution of time points for MAP infection correlated with infection progression. These patterns were not observed in the JD(+) macrophages, which presented a random pattern (Figure 1B). The signature of the transcriptome was clearly different in the JD(+) group. The analysis of the second and third principal components confirmed the general distinction between the two JD status groups (Figure 1C), suggesting a distinct response of JD(–) and JD(+) macrophages to MAP infection.

**Figure 2:**
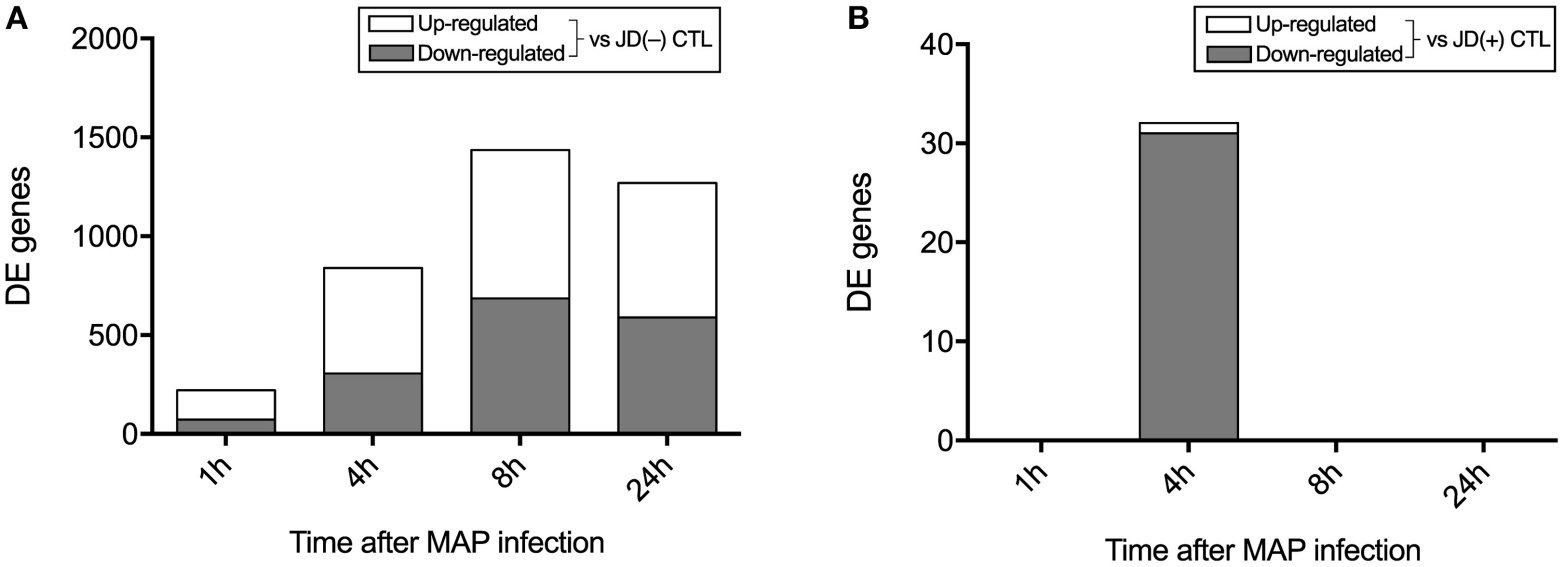
Number of differently expressed (DE) genes following MAP infection compared to the controls (CTL) in Johne's disease (JD) negative [JD(–)] macrophages **(A)** and JD positive [JD(+)] macrophages **(B)**.

Considering a significant *q* value threshold of 0.05 and fold changes ≥2, 1,533 genes were identified as DE in macrophages from JD(–) cows in response to MAP infection. Before *in vitro* infection, these macrophages had presumably not been exposed to MAP, since the cows were confirmed negative for JD during a survey period of 3–5 years. In these JD(–) macrophages, an important shift was observed as early as 1 hpi, with 221 DE genes, 148 upregulated and 73 downregulated, in response to MAP infection (Figure 2A). The number of DE genes increased as the infection progressed, with a total of 839, 1,436, and 1,269 DE genes at 4, 8, and 24 hpi, respectively. Upregulated and downregulated genes were in similar proportions. In primary JD(+) macrophages, the *in vitro* MAP infection had a minor effect on the transcriptome (Figure 2B). Only 32 genes were found to be significantly affected in the presence of live MAP, and that effect was observed only at 4 hpi. Incubation for 1, 8, or 24 h did not have a significant effect on gene expression of the six replicates in response to MAP infection. The effect of MAP on bovine macrophages was therefore more informative when JD(–) were analyzed, with a predictable trend of response to MAP infection that was not observed in JD(+) macrophages.

### MAP Affected the Expression of Genes Related to Specific Biological Processes in JD(–) Macrophages

#### Inflammatory Processes

Gram-positive bacteria such as MAP present pathogen-associated molecular patterns to members of the TLR family (29). In MAP-infected JD(–) macrophages, *TLR2* increased 2.1- to 3.1-fold (*q* ≤ 0.0147) throughout the 1-to-24-hpi period (Supplementary Table 4), while *TLR5* was distinctly downregulated (7.0-fold; *q* = 0.0042) at 4 hpi (Table 1) and remained negatively regulated at the 8 and 24 hpi period (Supplementary Table 4). *TLR8* expression only decreased later on at 24 hpi (Supplementary Table 4). It is well-known that MAP induces a pro-inflammatory program. Many pro-inflammatory genes were highly expressed during the first exposure of JD(–) macrophages to MAP infection. Tumor necrosis factor alpha (*TNF*), chemokine (CC), chemokine C-X-C motif ligand (CXCL), and interleukin (IL) genes, notably *CCL2–5, -8*, and *-10, CXCL2–3* and *-13, IL1A*, and *IL6*, were highly upregulated at several time points (Table 1, Supplementary Table 4). In addition, TNF receptor family members increased in response to MAP infection. The levels of TNF receptors *TNFRSF12A, TNFRSF18*, and *TNFRSF1B* increased from 4 hpi and remained DE throughout the 4–24 hpi period (Supplementary Table 4). The *TNFRSF6B* increased from 8 hpi and even later on up to 24 hpi (Supplementary Table 4). The expression of other immune regulating genes, including leukemia inhibitory factor (*LIF*), interleukin receptor-associated kinase 2 (*IRAK2*), oxidative stress responders such as prostaglandin-endoperoxide synthase 2 (*PTGS2*), hypoxia-inducible factor 1 subunit alpha (*HIF1*α), and nitric oxide synthase 2 (*NOS2*) (Table 1, Supplementary Table 4), were upregulated by MAP infection.

**Table 1:** Top 20 DE genes after MAP infection vs. uninfected CTL samples of macrophages from JD(–) cows.

**1 hpi**			**4 hpi**			**8 hpi**			**24 hpi**		
**Gene symbol**	**FC^a^**	***q*-value^b^**	**Gene symbol**	**FC^a^**	***q*-value^b^**	**Gene symbol**	**FC^a^**	***q*-value^b^**	**Gene symbol**	**FC^a^**	***q*-value^b^**
*IL6*	92.2	0.0027	*PTX3*	396.7	0.0007	*PTX3*	344.1	0.0005	*EDN1*	145.7	0.0005
*IL1A*	57.1	0.0027	*EDN1*	216.8	0.0007	*EDN1*	264.5	0.0005	*IL6*	132.8	0.0005
*PTX3*	54.2	0.0027	*IL6*	153.4	0.0007	*IL6*	175.5	0.0005	*CSF2*	92.7	0.0005
*EDN1*	47.4	0.0027	*TNF*	124.2	0.0007	*CSF2*	154.2	0.0005	*CTSL*	75.4	0.0005
*F3*	44.3	0.0027	*CSF2*	123.8	0.0007	*LIF*	127.6	0.0023	*WFDC18*	70.6	0.0005
*TNF*	42.3	0.0027	*LIF*	112.5	0.0007	*CCL20*	127.5	0.0005	*IL1A*	60.4	0.0005
*NR4A1*	34.4	0.0027	*F3*	85.4	0.0007	*F3*	97.2	0.0005	*F3*	57.7	0.0005
*CCL8*	32.8	0.0027	*CCL8*	55.5	0.0007	*CSF3*	79.5	0.0005	*KCNJ15*	56.8	0.0005
*LIF*	28.3	0.0131	*IL1A*	55.4	0.0007	*TNF*	73.5	0.0005	*MT1A*	56.4	0.0005
*RND1*	26.8	0.0027	*CCL4*	51.5	0.0007	*FGF18*	70.6	0.0256	*CSF3*	54.6	0.0005
*MMP2*	−24.5	0.0027	*SLC40A1*	0-6.7	0.0007	*EHHADH*	−23.0	0.0005	*MMP2*	−40.5	0.0027
*SPARC*	−38.0	0.0027	*TFAP4*	−6.8	0.0156	*TNC*	−26.6	0.0005	*ALOX5*	−48.0	0.0009
*ACTA2*	−41.1	0.0311	*EHHADH*	−6.9	0.0007	*SLC40A1*	−27.0	0.0005	*GJA1*	−48.7	0.0005
*TAGLN*	−82.6	0.0131	*TLR5*	−7.0	0.0042	*COL3A1*	−27.4	0.0005	*S1PR1*	−55.6	0.0005
*COL3A1*	−87.5	0.0027	*S1PR1*	−7.4	0.0007	*COL1A2*	−28.6	0.0005	*FBN1*	−61.1	0.0005
*COL6A2*	−93.3	0.0297	*PDK4*	−7.6	0.0007	*COL1A1*	−29.9	0.0005	*SLC1A3*	−66.8	0.0009
*COL1A2*	−110.6	0.0027	*FLNC*	−8.9	0.0171	*S1PR1*	−33.5	0.0005	*SPIC*	−97.6	0.0062
*COL6A1*	−113.7	0.0297	*ANKRD1*	−12.1	0.0257	*COL6A2*	−33.6	0.0097	*SPARC*	−199.6	0.0062
*TNC*	−148.5	0.0047	*PIK3IP1*	−14.8	0.0007	*COL6A1*	−34.2	0.0040	*COL1A1*	−433.2	0.0487
*COL1A1*	−258.4	0.0196	*GSTM3*	−18.7	0.0072	*PIK3IP1*	−44.7	0.0005	*COL1A2*	−524.6	0.0051

a *Fold change (FC) values are the ratio (log2) of FPKM values of MAP-infected MDMs vs. uninfected controls (CTL) from JD(–) cows. A negative fold change reports downregulated gene expression*.

b *FDR-corrected q-value*.

Other genes known to be implicated in the resolution of inflammation were also identified as being significantly upregulated, including genes encoding anti-inflammatory cytokines, or mediators of, such as *IL10, IL33, BCL3, CISH* (cytokine-inducible SH2-containing protein), *SOCS3* (suppressor of cytokine signaling 3), *TRAF1* (TNF receptor-associated factor 1), *TNFAIP3* (TNF alpha-induced protein 3), *TNIP1* (TNFAIP3-interacting protein 1), *TNIP2, TNFAIP6, NFKB1* (nuclear factor kappa B [NF-κβ] subunit 1), *NFKBB* (NF-κβ inhibitor beta), *MAP3K8* (mitogen-activated protein 3 kinase 8), *IRAK3*, and *ATF3* (activating transcription factor 3) (Supplementary Table 4). The negative regulator of NF-κB gene promoters, *ATF3*, was already greatly induced by MAP at 1 hpi (5.1-fold) and also for the entire infection period (Supplementary Table 4). ATF3 is the transcriptional inhibitor of tenascin-C (*TNC*) (43). Supporting the *TNC* downregulation effect of ATF3, *TNC* was one of the most downregulated genes by MAP infection, being reduced 148 times at 1 hpi in MAP-infected JD(–) macrophages (Table 1). Both anti- and pro-inflammatory DE genes were confirmed by qPCR, notably *IL1A, IL6, IL10, CCL2, SOCS3*, and *TNFAIP6* (Table 2). Taken together, these results show that both pro- and anti-inflammatory gene expressions were observed, suggesting an initial concomitant pro- and anti-inflammatory reaction at the beginning of the infection in JD(–) macrophages.

**Table 2:** RNA-seq analysis and validation by qPCR of DE genes from MAP-infected macrophages.

**Gene symbol**	**JD(–) at 8 hpi vs. CTL JD(–)**				**JD(+) at 8 hpi vs. CTL JD(+)**				**CTL JD(+)/CTL JD(–)**			
	**RNA-seq (*****n*** **=** **6)**		**qPCR (*****n*** **=** **18)**		**RNA-seq (*****n*** **=** **6)**		**qPCR (*****n*** **=** **19)**		**RNA-seq (*****n*** **=** **6/6)**		**qPCR (*****n*** **=** **19/18)**	
	**FC^a^**	***q*-value**	**FC^b^**	***p*-value**	**FC^a^**	***q*-value**	**FC^b^**	***p*-value**	**FC^c^**	***q*-value**	**FC^d^**	***p*-value**
*ABCA1*	1.1	0.7320	1.1	0.9341	1.4	0.9999	−1.3	0.0135	−1.2	0.6503	1.2	0.3690
*ABCA7*	−2.1	0.0133	−2.2	0.0002	−1.1	0.9999	−2.4	<0.0001	−2.5	0.0268	1.2	0.6516
*ABCA9*	−3.7	0.0005	−14.9	0.0001	−2.5	0.6510	−8.3	0.0007	−2.3	0.0217	−3.1	0.0042
*ABCG1*	−1.1	0.9541	1.3	0.5340	1.5	1.0000	−2.6	0.0324	−6.2	0.0745	2.0	0.4502
*ARG2*	5.6	0.0005	4.1	0.0001	2.0	0.9823	1.5	0.0020	1.5	0.4285	3.0	<0.0001
*CCL2*	7.6	0.0005	18.4	<0.0001	−1.4	0.9999	2.1	0.1278	2.2	0.0886	5.6	<0.0001
*CSF3*	79.5	0.0005	70.9	<0.0001	3.0	0.9673	4.1	0.0001	3.1	0.1253	18.8	<0.0001
*CYP27A1*	−4.0	0.0005	−2.3	0.0009	−1.4	0.9999	−1.9	0.002	−2.9	0.0018	−1.2	0.0001
*GAS7*	−1.2	0.3280	−2.1	0.0052	−1.3	0.9999	−5.2	0.0005	−2.0	0.0225	2.2	0.2078
*GPD2*	3.3	0.0005	3.5	0.0001	1.2	0.9999	2.8	0.0002	2.0	0.0185	2.2	0.0026
*GPX4*	1.2	0.3496	1.3	0.0402	1.6	0.9893	1.0	0.1526	−2.7	0.0018	−1.2	0.2609
*GSR*	−2.3	0.0005	1.2	0.4831	1.6	0.9999	1.0	0.0914	−1.6	0.1739	−1.1	0.9033
*HIF1A*	2.8	0.0019	3.0	<0.0001	−1.3	0.9999	1.5	0.0033	2.2	0.0330	2.4	<0.0001
*IDO1*	55.5	0.0005	125.5	<0.0001	1.8	0.9999	5.0	0.0152	1.6	0.3977	40.0	0.0193
*IL10*	8.8	0.0005	4.0	<0.0001	−1.3	0.9999	1.1	0.0030	3.9	0.0018	2.5	0.0002
*IL1A*	66.2	0.0005	130.6	<0.0001	1.5	0.9999	4.1	0.0001	8.0	0.0018	31.4	<0.0001
*IL33*	8.2	0.0005	5.1	0.0676	−2.2	0.9999	−1.8	0.3396	7.8	0.0163	6.2	0.7550
*IL6*	175.5	0.0005	55.4	0.0010	1.1	0.9999	5.7	0.0002	31.6	0.0018	7.4	0.0138
*NOS2*	64.2	0.0005	44.4	<0.0001	2.8	0.8084	7.9	<0.0001	8.7	0.0018	9.8	<0.0001
*PPARG*	−4.8	0.0023	−3.7	0.0067	−1.5	0.9999	−1.8	0.0067	−1.9	0.1030	1.1	0.0001
*NR1H3 (LXRα)*	1.3	0.2776	1.4	0.0001	1.2	0.9999	−1.5	0.0001	1.0	0.9039	2.2	0.0032
*NR1H4 (RXR)*	−4.4	0.0344	−8.6	0.0039	−1.9	1.0000	−3.9	0.0011	−1.7	0.4365	−2.6	0.0344
*NRIP1 (RIP140)*	*n*.*a*.	n.a.	−1.8	0.0084	*n*.*a*.	n.a.	−1.3	0.0215	*n*.*a*.	n.a.	−1.4	0.2694
*PTGS2*	21.3	0.0005	18.1	0.0039	2.5	0.6906	5.1	0.0006	2.5	0.0708	8.3	0.0032
*PTX3*	344.1	0.0005	35.0	0.0020	5.7	0.3513	8.8	0.0005	19.8	0.0018	26.7	0.0015
*SERPINE1*	−1.1	0.7813	2.9	0.0046	−2.0	0.9999	−1.1	0.0266	11.3	0.0032	1.8	0.2116
*SLC39A14*	9.3	0.0005	2.6	0.2188	−2.0	0.9999	−1.1	0.0781	5.6	0.0018	1.2	0.7095
*SOCS3*	7.9	0.0005	14.5	0.0001	3.0	0.4989	3.2	0.0002	−1.8	0.2378	5.8	0.0018
*SPINT2*	−1.1	0.8910	1.1	0.8077	−1.0	0.9999	−1.0	0.6257	−2.2	0.0477	−1.8	0.0093
*TNFAIP6*	33.0	0.0005	19.6	0.0002	−1.4	0.9999	1.2	0.7148	5.8	0.0333	37.0	0.0002

a *Fold change values are the ratio of FPKM values of MAP-infected macrophages vs. non-infected CTL macrophages. A negative fold change value reports downregulated gene expression. The number of replicates were n = 18 for JD(–D cow and n = 19 for JD(+) cows*.

b *Fold change values are the ratio of normalized values obtained by qPCR of MAP-infected macrophages versus CTL macrophages. The log2 fold values are reported as fold change values. A negative fold change reports downregulated gene expression. Number of replicates were n = 6 for both JD groups*.

c *Fold change values are the ratio of FPKM values of CTL JD(+) macrophages vs. non-infected CTL JD(–) macrophages. A negative fold change value reports downregulated gene expression. The number of replicates were n = 18 for JD(–D cows and n = 19 for JD(+) cows*.

d *Fold change values are the ratio of normalized values obtained by qPCR of MAP-infected macrophages versus CTL macrophages. The log2 fold values are reported as fold-change values. A negative fold-change reports down-regulated gene expression. The number of replicates were n = 19 for JD(+) cows and n = 18 for JD(−) cows*.

*n.a., not available (i.e., not detected by RNA-seq analysis)*.

#### Macrophage Polarization

The plasticity of macrophages is well-known, and polarization markers reflect the transition of macrophages to different phenotypes (24). Both M1 and M2 polarization markers were DE in JD(–) macrophages in response to MAP infection. Classically activated M1 macrophages are phenotypically characterized by M1 markers such as CD80. During the infection of JD(–) macrophages, *CD80* expression was already DE at 1 hpi and progressively increased during the 24-h period (Supplementary Table 4). Also, these macrophages were metabolically associated with glycolysis activity.

The glucose transporter encoding genes were also significantly affected by MAP infection. The solute carriers 2 (SLC2) genes *SLC2A1, SLC2A3*, and *SCL2A6* were all upregulated at 4 hpi and remained overexpressed until 24 hpi (Supplementary Table 4). The *PFKFB3* gene, another gene known for promoting cell glycolysis (44), was transiently upregulated at 4- and 8-hpi time points (Supplementary Table 4). Metabolic reprogramming is required for appropriate glucose flux during macrophage polarization (45). The carbohydrate kinase-like protein (CARKL) controls the cellular metabolism like a rheostat on the pentose phosphate pathway, which is an alternative to glycolysis (46). In our study, *CARKL* was downregulated from 4 until 24 hpi in MAP-infected JD(–) macrophages (Supplementary Table 4). While arginine metabolism via arginase is at the center of the M1/M2 polarization of macrophages (47), the arginase gene 1 (*ARG1*) was marginally abundant and not affected by MAP infection (Supplementary Table 4). The arginase gene 2 (*ARG2*), however, was promptly upregulated at 1 hpi and further increased later on during the 4-to-24-hpi period. The scavenger receptor CD163, a marker of the alternative activation pathway, was reduced only at a later time (24 hpi, Supplementary Table 4). In contrast, genes associated to M2 polarization, such as Krüppel-like factor 4 (*KLF4*) and *IL10*, were upregulated at the early time. *KLF4* was found transiently upregulated at 1 and 4 hpi while *IL10* was drastically increased by ~20-fold at 1 hpi (Supplementary Table 4). *IL10* declined later on but remained DE at 24 hpi (Supplementary Table 4). The 4.0-fold change of *IL10* at 8 hpi was confirmed by qPCR (*p* < 0.0001; Table 2). The gene expression of protein arginine methyltransferase 1 (*PRMT1*), whose activity is necessary for the proto-oncogene c-Myc function in M2 macrophage differentiation (48), and MYC was slightly increased at 8 hpi (2.0- and 3.2-fold change, respectively) in MAP-infected JD(–) macrophages (Supplementary Table 4). Overall, some M1 and M2 marker genes were upregulated by MAP, while a dominant metabolic profile promoting glycolysis seemed to prevail during the early stage of the infection.

#### Intracellular Transport

In mycobacteria-infected cells, the prevention of fusion between phagosomes and lysosomes contributes to mycobacterial survival. A tryptophan aspartate-containing coat protein, CORO1A is present on the phagosomal membrane and prevents lysosomal fusion. The expression of *CORO1A* was upregulated from 4 hpi and reached 4.0-fold at 24 hpi (Supplementary Table 4). A gene encoding the rate-limiting enzyme of tryptophan metabolism, indoleamine 2,3-dioxygenase (*IDO1*), was confirmed by qPCR to be 125.5-fold increased at 8 hpi (*p* < 0.0001; Table 2). It remained DE at 24 hpi (15.1-fold; Supplementary Table 4). Other genes encoding nascent autophagosome inhibitors or regulators of macroendosome biogenesis were upregulated in MAP-infected JD(–) macrophages, notably *RUBCNL* from 1 hpi and throughout the infection period (Supplementary Table 4). The p53-inducible protein 1 (TP53INP1) promotes autophagy by interacting with autophagosome-localized autophagy-related protein family (ATG) proteins (49). Even though *ATG4D* expression was slightly (~2-fold) sustained by 4 hpi and throughout the infection period, *TP53INP1* was repressed by 2.6-fold at 8 hpi (Supplementary Table 4). Interestingly, the solute carrier (*SLC*) genes *SLC1A3, SLC2A5, SLC40A1*, and *SLC46A3*, which encode, respectively, glutamate transporter, glucose/fructose transporter, iron transporter, and carrier of catabolites across the lysosomal membrane, were all downregulated during the 8-to-24-hpi period in JD(–) macrophages (Supplementary Table 4). Only the expression of divalent metal transporter *SLC39A14* increased during MAP infection (Supplementary Table 4). With those two exceptions, negative regulation of SLC genes can promote host survival by limiting access of MAP to nutrients in intracellular compartments and by autophagy.

#### Enriched DE-Genes Associated to Microbial Recognition Receptor Pathways

Analysis of canonical signaling DE genes revealed the top enriched pathways at 8 hpi (Table 3), notably in the inflammatory signaling pathways, namely, IL6, IL10, TNF, CD40 (cluster of differentiation 40), and TREM1 (triggering receptor expressed on myeloid cells 1). The top 30 most enriched pathways at other time points are reported in Supplementary Tables 5–8. The TREM1 and TLR pathways present similar DE genes. The *TREM1* was increased by 4 hpi and remained overexpressed later on at 24 hpi in JD(–) macrophages (Supplementary Table 4). Those pathways exerted synergistic effects (i.e., positive IPA *z* score; Table 3, Supplementary Tables 5–8). Microbial recognition triggers the formation of the inflammasome complex that involves specific nucleotide oligomerization domain (NOD)-like receptors (NLR) of the TREM1 and TLR pathways (50). *NLRP3, NLRP12*, and *NLRC4* were upregulated at 4 hpi and remained highly expressed up to 24 hpi (Supplementary Table 4). These NLR members contain carboxy-terminal leucine-rich repeats similar to NOD1 and NOD2, the intracellular sensors of bacterial pathogens (51). While these NLR members were upregulated, *NOD1* was downregulated from 4 to 24 hpi, whereas *NOD2* remained unchanged in response to MAP (Supplementary Table 4). Upon activation, NLRs or NOD1/2 recruit the receptor-interacting serine/threonine kinase 2 (RIPK2) (51). *RIPK2* was transiently upregulated from 4 to 8 hpi (Supplementary Table 4). RIPK2, which is the cornerstone of the NLR pathway, is a potent activator of NF-κB cascade (52).

**Table 3:** Top 30 IPA pathways enriched by DE genes at 8 hpi in macrophages from JD(–) cows.

**Pathways**	**Adj. *p*-value^a^**	**Number of genes^b^**	***z* score^c^**
Hepatic fibrosis/Hepatic stellate cell activation	3.16E−11	43	n.a.
Role of macrophages, fibroblasts and endothelial cells in rheumatoid arthritis	3.16E−11	57	n.a.
IL-10 signaling	3.98E−09	23	n.a.
Atherosclerosis signaling	7.76E−09	31	n.a.
TREM1 signaling	2.24E−08	23	2.00
IL-6 signaling	1.02E−07	28	3.27
Production of nitric oxide and reactive oxygen species in macrophages	1.10E−07	36	2.00
Valine degradation I	1.26E−07	11	n.a.
Role of IL-17A in arthritis	2.57E−07	18	n.a.
Virus entry via endocytic pathways	4.57E−07	23	n.a.
TNFR2 signaling	4.68E−07	13	1.90
LXR/RXR activation	6.31E−07	27	−2.86
Caveolar-mediated endocytosis signaling	6.46E−07	20	n.a.
Death receptor signaling	6.46E−07	23	1.04
CD40 signaling	6.76E−07	19	0.00
Cholecystokinin/Gastrin-mediated signaling	8.13E−07	24	2.04
PPAR signaling	8.32E−07	23	−3.96
Toll-like receptor signaling	9.77E−07	20	2.14
IL-12 signaling and production in macrophages	9.77E−07	28	n.a.
Differential regulation of cytokine production in macrophages and T helper cells by IL-17A and IL-17F	1.00E−06	10	n.a.
Acute phase response signaling	1.15E−06	32	2.65
HMGB1 signaling	1.20E−06	26	1.96
Differential regulation of cytokine production in intestinal epithelial cells by IL-17A and IL-17F	1.38E−06	11	n.a.
IL-8 signaling	2.45E−06	33	2.04
Glutaryl-CoA degradation	2.75E−06	8	n.a.
Role of tissue factor in cancer	2.88E−06	24	n.a.
Folate transformations I	3.16E−06	7	n.a.
Altered T cell and B cell signaling in rheumatoid arthritis	3.16E−06	21	n.a.
Induction of apoptosis by HIV1	3.24E−06	17	0.50
Agranulocyte adhesion and diapedesis	3.72E−06	33	n.a.

a *The Benjamini–Hochberg multiple testing adjusted p-value*.

b *The DE genes associated with their respective pathway are listed in the Supplementary Table 7*.

c *IPA activation score (positive value, activated pathway; negative value, inhibited pathway). n.a., not available*.

#### DE-Gene-Enriched Chronic Disease Related Pathways

In the functional analysis, IPA pathways were identified at all infection periods. The “Role of macrophages, fibroblasts and endothelial cells in rheumatoid arthritis” pathway was top-ranked from 4 hpi (Supplementary Table 6) and included the highest number of DE genes at the 8-hpi time point (Table 3, Supplementary Table 7, Supplementary Figure 3). The “Hepatic fibrosis/hepatic stellate cell activation” pathway was also constantly among the top-ranked pathways. These two pathways ranked first at all other infection time points (Supplementary Tables 5–8). DE genes reported in both pathways in MAP-infected JD(–) macrophages were notably pro-inflammatory (e.g., *CCL2, CCL5, IL1A*, and *IL6*), anti-inflammatory (e.g., *SOCS3, NFKB1* [p50], and *IL10*), anti-apoptotic (e.g., *TNFRSF1B*), and extracellular matrix remodeling or pro-fibrotic genes (e.g., *MMP13, PDGFA*, and *PDGFB*). Most of the pro-fibrotic genes were associated with the “Hepatic fibrosis/hepatic stellate cell activation” pathway (Supplementary Figure 5), with the exception of *COL12A1* and *EDN*-*1*, the latter being among the top upregulated genes (Table 1). Many collagen alpha-chain protein genes, notably *COL1A1, COL1A2, COL6A1, COL6A2*, and *CTGF*, were downregulated by MAP (Supplementary Tables 4, 8). They represented half of the top 10 downregulated DE genes at 1 and 8 hpi in JD(–) macrophages (Table 1). Most of the genes that enriched pathways such as the “Hepatic fibrosis/hepatic stellate cell activation” pathway were downregulated (Supplementary Table 8, Supplementary Figure 5A). In contrast, the genes that enriched the “Role of macrophages, fibroblasts and endothelial cells in rheumatoid arthritis” pathway were upregulated (Table 1, Supplementary Tables 5–8). Overall, numerous NF-κβ pathway members involving pro- and anti-inflammatory signals were upregulated. Additionally, in the functional analysis, significant pathways associated with IL-17 were identified by IPA software, notably the “Differential regulation of cytokine production in macrophages by IL-17A and IL-17F” pathway, the “Differential regulation of cytokine production in intestinal epithelial cells by IL-17A and IL-17F” pathway, and the “Role of IL-17A in arthritis” pathway at 4 hpi (Supplementary Table 6) and during other MAP-infection periods (Supplementary Tables 5, 7, 8). Among others, the TLR, CD40, TREM, iNOS, and IL10 signaling pathways were identified in MAP-infected JD(–) macrophages. Defective inflammation resolution is the underlying cause of prevalent chronic inflammatory diseases, notably arthritis, rheumatitis, and atherosclerosis diseases. It is noteworthy that these putative pathways driving these diseases were also associated to paratuberculosis in this study.

### JD(+) Macrophages Were Less Responsive to MAP Infection

Macrophages from infected JD(+) animals did not show a clear response when exposed *in vitro* to live MAP challenge (Figure 2B). Only 32 significantly DE genes (*q* ≤ 0.05) were identified with a biologically relevant expression threshold (≥2-fold change, Table 4). Most of the significant DE genes were downregulated at 4 hpi in JD(+) macrophages. The inflammatory marker pentraxin 3 (*PTX3*) was upregulated in JD(+) macrophages at 4 hpi (Table 4), which was confirmed by qPCR at 8 hpi (Table 2). Interestingly, CTL macrophages (i.e., not infected) from JD(+) cows expressed already ~20 times more *PTX3* than the CTL JD(–) macrophages did (Supplementary Table 4), a result confirmed by qPCR (26.7-fold; Table 2). In addition, the repression of *NR1H3* (encoding LXRα), which is known to be downregulated by PTX3 (53), was confirmed using qPCR to be lower expressed in CTL JD(+) in comparison with CTL JD(–) macrophages. A number of cytokines, including TNFα, induce the extracellular matrix glycoprotein, *TNC*, associated with the pathogenesis of atherosclerosis and foam cell formation (54). Both *TNF* and *TNC* level in CTL JD(+) macrophages was upregulated in comparison to JD(–) macrophages (Supplementary Table 4).

**Table 4:** Top 20 DE genes at 4 hpi in JD(+) macrophages.

**Gene symbol**	**FC^a^**	***q*-value^b^**
*PTX3*	20.7	0.0419
*COL1A2*	−39.9	0.0333
*NT5E*	−40.5	0.0481
*COL3A1*	−42.7	0.0206
*SERPINE2*	−45.0	0.0206
*LGR4*	−46.8	0.0333
*FBN1*	−48.3	0.0206
*NID2*	−52.0	0.0206
*NES*	−53.5	0.0206
*GJA1*	−56.8	0.0206
*LAMA4*	−57.5	0.0481
*SFRP4*	−58.7	0.0333
*LUM*	−58.9	0.0333
*CDH11*	−64.4	0.0206
*DCN*	−78.2	0.0206
*PDGFRA*	−82.5	0.0419
*CAV1*	−101.4	0.0206
*COL1A1*	−124.9	0.0206
*PTN*	−127.3	0.0206
*TNFSF18*	−197.8	0.0206

a *Fold change (FC) values are the FPKM ratio of MAP-infected vs. CTL macrophages from JD(+) cows. A negative fold change reports downregulated gene expression*.

b *FDR-corrected q-value*.

While MAP infection did not have much impact on JD(+) macrophages, the basal expression of several genes in the unchallenged (CTL) JD(+) macrophages was more elevated in comparison with CTL JD(–) macrophages. The comparison of CTL samples from both JD groups showed 868 DE genes, with the top DE genes shown in Table 5. This difference was confirmed using qPCR for *CCL2, CSF3, IL10, IL6, H1F1A, NOS2*, and *IL1A* with 37 animals (Table 2). This phenomenon was not generalized but rather gene-specific, since other genes like *CYP27A1, GAS7, GPX4, GSR, PPARG, SERPINE1, SLC39A14, ABCA1, ABCG1*, and *NRIP1* were not confirmed DE by qPCR (Table 2).

**Table 5:** Top 20 DE genes between CTL macrophages from JD(+) cows vs. JD(–) cows.

**Gene symbol**	**FC^a^**	***q*-value^b^**
*FAP*	313.4	0.0073
*SFRP4*	202.1	0.0018
*COL11A1*	190.9	0.0352
*FGF7*	161.1	0.0054
*LUM*	157.4	0.0018
*NOV*	153.3	0.0032
*IL1RL1*	148.1	0.0201
*C1QTNF3*	145.6	0.0018
*SGCE*	137.3	0.0195
*PTN*	127.1	0.0018
*MATK*	−4.7	0.0018
*FAM195A*	−4.7	0.0018
*HCRTR1*	−5.1	0.0284
*DNM1*	−5.7	0.0054
*TRIM47*	−6.1	0.0018
*ELMO3*	−6.6	0.0352
*FABP4*	−7.4	0.0018
*SGCA*	−7.9	0.0018
*EEF1A2*	−9.1	0.0018
*OOSP1*	−12.0	0.0144

a *Fold change (FC) values are the ratio of FPKM values of JD(+) CTL macrophages vs. JD(–) CTL macrophages. A negative fold change reports downregulated gene expression*.

b *FDR corrected q-value*.

The comparison of CTL samples from both JD groups showed several pathways affected by JD status (Table 6). Moreover, they were found to be similar to the ones affected by 24 hpi in JD(–) macrophages (Supplementary Table 8); i.e., the DE genes associated with those pathways were also similar (Supplementary Tables 8 vs. 9). Although JD(+) macrophages are less responsive to MAP infection than JD(–) macrophages, a signature of chronic disease-related pathways was observed in both groups at 4 hpi (Supplementary Tables 6, 10, respectively). Interestingly, these pathways, namely, the “Role of macrophages in rheumatoid arthritis,” “Hepatic fibrosis,” and “Atherosclerosis” signaling, were also identified when CTL JD(+) macrophages were compared with CTL JD(–) macrophages (Supplementary Table 9). Overall, JD(+) macrophages were less responsive to MAP infection than JD(–) macrophages, suggesting that the transcriptomic profile of JD(+) macrophages was already formatted prior to *in vitro* infection.

**Table 6:** Top 30 IPA pathways enriched by DE genes between CTL JD(+) and CTL JD(–) macrophages.

**Pathways**	**Adj. *p*-value^a^**	**Number of genes^b^**	***z* score^c^**
Hepatic fibrosis/Hepatic stellate cell activation	7.94E−14	37	n.a.
Semaphorin signaling in neurons	2.45E−05	13	n.a.
Role of macrophages, fibroblasts, and endothelial cells in rheumatoid arthritis	4.57E−05	32	n.a.
Granulocyte adhesion and diapedesis	2.45E−04	22	n.a.
Inhibition of matrix metalloproteases	1.55E−03	9	n.a.
LXR/RXR activation	1.66E−03	16	−0.58
Axonal guidance signaling	1.66E−03	36	n.a.
Role of tissue factor in cancer	1.70E−03	15	n.a.
Acute phase response signaling	2.24E−03	19	2.18
ILK signaling	2.40E−03	20	2.52
Agranulocyte adhesion and diapedesis	2.69E−03	20	n.a.
Colorectal cancer metastasis signaling	2.69E−03	23	2.13
HMGB1 signaling	2.88E−03	15	1.07
Intrinsic prothrombin activation pathway	3.63E−03	7	n.a.
Atherosclerosis signaling	3.63E−03	15	n.a.
TREM1 signaling	4.90E−03	11	n.a.
IL-6 signaling	4.90E−03	14	2.14
Ephrin receptor signaling	4.90E−03	18	n.a.
Role of osteoblasts, osteoclasts, and chondrocytes in rheumatoid arthritis	4.90E−03	21	n.a.
Differential regulation of cytokine production in intestinal epithelial cells by IL-17A and IL-17F	5.01E−03	6	n.a.
Folate transformations I	5.75E−03	4	n.a.
IL-10 signaling	6.92E−03	10	n.a.
Coagulation system	7.76E−03	7	1.13
Renal cell carcinoma signaling	8.32E−03	10	0.82
Chemokine signaling	8.32E−03	10	1.27
Signaling by rho family GTPases	8.32E−03	21	−0.24
Superpathway of cholesterol biosynthesis	1.15E−02	6	n.a.
Altered T cell and B cell signaling in rheumatoid arthritis	1.15E−02	11	n.a.
HIF1α signaling	1.15E−02	12	n.a.
CCR3 signaling in eosinophils	1.17E−02	13	n.a.

a *The Benjamini–Hochberg multiple testing adjusted p-value*.

b *The DE genes associated with pathways are listed in Supplementary Table 9*.

c *PA activation score (positive value, activated pathway; negative value, inhibited pathway)*.

### Expression of Putative Tolerized Genes Was Promptly Induced in MAP-Infected JD(–) Macrophages

The NF-κB related genes *NFKBIA* (alias IκBα) and *TNFAIP3* (encoding A20 protein) play critical roles in termination of the canonical NF-κB pathway (55). Those genes were significantly upregulated at all time points in JD(–) macrophages in response to MAP exposure, presumably at the first exposure to MAP since macrophages were from J(–) cows. It is well-known that endotoxin-tolerant cells increase the expression of negative NF-κB regulators, notably *TNFAIP3, NFKB1* (encoding p50), NF-κB inhibitor Zeta (*NFKBIZ*), *IRAK3*/IRAK-M, and the suppressor of cytokine signaling (SOCS) genes (56), among others. Those factors are part of a classical negative feedback system that regulates cytokine signal transduction through a so-called tolerization mechanism. These tolerized genes were significantly upregulated in JD(–) macrophages in response to first MAP exposure. The expression of *SOCS3* was promptly upregulated at 1 hpi and increased to 8.9-fold at 24 hpi (Supplementary Table 4). The upregulation of SOC3 was confirmed DE at 8 hpi by qPCR (Table 2). Other negative regulators, notably the monocyte chemoattractant protein 1 gene (*CCL2)* and the granulocyte colony-stimulating factor (known as G-CSF) gene (*CSF3*), were upregulated at 8 hpi (Table 2). The response to MAP infection was prompt and sustained in JD(–) macrophages for tolerizable NF-κβ target genes such as *IL10, CCL2, CSF3, IDO1*, and *NOS2*, whereas slight or no changes were detected in JD(+) macrophages for *CCL2, NOS2*, and *IL1A* (Table 2). Genes that are not NF-κβ targets, such as *CYP27A1, GAS7, GPX4, GSR, PPARG, SERPINE1, ABCA1, ABCA7, ABCA9, ABCG1*, and *NRIP1*, were not upregulated in MAP-infected JD(–) macrophages (Table 2).

Globally, whereas few DE genes were observed in JD(+) macrophages, the magnitude of the response to MAP infection was always much more considerable in macrophages from JD(–) compared to JD(+). For example, at 8 hpi, *NOS2* and *IDO1* were upregulated in both JD(+) and JD(–) macrophages but at a higher degree in the latter (7.9- vs. 44.4-fold change, respectively; *p* < 0.0001) (Table 2). Other genes were affected in the same way, notably the oxidative stress marker *NOS2*, which is one of the tolerized immune genes (56). NOS2 is also part of the “Role of macrophages in rheumatoid arthritis” (Supplementary Figure 4) and “LXR/RXR activation” pathways (Supplementary Figure 6).

### Putative Epigenetic Modifiers in the Second Wave of Gene Expression Following MAP Infection

During the initial “cytokine storm” (section inflammatory processes), the gene expression of DNA methyltransferase 1 (*DNMT1*), responsible for gene hypermethylation, increased ~2-fold at 24 hpi (Supplementary Table 4). The growth arrest and DNA-damage-inducible protein 45 alpha (*GADD45A*) and gamma (*GADD45G*) genes are members of a small family of stress-response genes and serve as a nexus between DNA repair and epigenetics (57). The *GADD45A* and *GADD45G* genes were downregulated from 4 hpi until 24 hpi in MAP-infected JD(–) macrophages (Supplementary Table 4). Another epigenetics-related gene, encoding lysyl oxidase-like 2 (*LOXL2*), was downregulated in JD(–) macrophages by −20.6-fold change as early as 1 hpi, while in CTL JD(+) macrophages, in comparison with CTL JD(–) macrophages, *LOXL2* was upregulated ~10-fold (Supplementary Table 4). This transcriptional corepressor specifically mediates the deamination of trimethylated Lys-4 of histone H3 (*H3K4me3*), a specific tag for epigenetic transcriptional activation (58). Histone deacetylases (HDACs) are other key participants in epigenetic regulation of immune responses. The *HDAC6* gene, involved in the HDAC6/STAT3/IL10 axis (59), was downregulated ~3-fold later on (24 hpi) during infection in JD(–) macrophages (Supplementary Table 4). In parallel, *IL10* expression that was drastically upregulated ~20-fold at 1 hpi was progressively reduced to a 3.7-fold change at 24 hpi (Supplementary Table 4). The change in these epigenetic modifiers during MAP infection suggests that gene expression is also regulated at the epigenetic level.

### MAP Affected Expression of Genes Involved in Lipid Homeostasis and Accumulated Lipids in JD(+) Macrophages

#### Lipid-Mediated Immunosuppressive Effect

In response to mycobacterial infection, the TLR-mediated signaling cascade releases arachidonic acid by the action of phospholipases (60). In JD(–) macrophages, genes encoding phospholipases, notably LDL-associated phospholipase A2 (*PLA2G7*), phosphatidylcholine 2-acylhydrolase 12A (*PLA2G12A*), and lysosomal phospholipase A2 (*PLA2G15*), were all downregulated at 8 and 24 hpi in response to MAP infection (Supplementary Table 4). Eicosanoids are normally metabolized into either leukotriene by the activity of arachidonate 5-lipoxygenase (ALOX5) or prostaglandin (PGE2) by the activity of cyclooxygenase (COX2) (61). In our results, *ALOX5* was drastically downregulated (−12- to 48-fold), whereas the inducible COX2 encoding gene, *PTGS2*, increased (47- to 21-fold) during the 8-to-24-hpi period (Supplementary Table 4). The COX2 enzyme catalyzes the rate-limiting step of inflammatory production of bioactive lipid PGE2 that plays important immunosuppressive roles (60), notably by modulating the gene expression of program cell death ligand-1 (*CD274*) (62). The expression of *CD274* increased transiently (4–8 hpi) ~2-fold in response to MAP infection in JD(–) macrophages (Supplementary Table 4). The S100A8/9 proteins are members of the S100 gene family of calcium-binding proteins and are also known to function as fatty acid carriers. Both S100A8/9 were upregulated at 8 hpi (Supplementary Table 4). Downregulation of the growth arrest-specific gene 7 (*GAS7*) has been associated with chronic inflammation and obesity (63). The qPCR results showed that *GAS7* was downregulated at 8 hpi in MAP-infected JD(+) macrophages (Table 2).

#### Lipid Synthesis

Lipid homeostasis pathways, including PPAR signaling, LXR/RXR activation, and hepatic cholestasis pathways, were gradually altered during MAP infection. The LXR/RXR activation pathway moved from the 39th most impacted pathway at 1 hpi to the 35th, 12th, and 3rd positions at 4, 8, and 24 hpi, respectively in JD(–) macrophages, suggesting an increased impact on cholesterol efflux and lipogenesis pathways as the infection progressed (Supplementary Tables 5–8). It is worth mentioning that the LXR/RXR activation pathway ranked 6th in CTL JD(+) macrophages in comparison with CTL JD(–) macrophages (Table 6). Their negative IPA *z* score value suggest that CTL JD(+) macrophages strongly repressed these pathways. The RXR gene *NR1H4*, which is the obligate partner of *LXR*, was confirmed to be downregulated −2.6-fold in CTL JD(+) macrophages (*p* = 0.0344) (Table 2) and even more downregulated in response to MAP infection in JD(–) macrophages (Table 2). In particular, the transcriptional activator required for lipid homeostasis, the gene encoding the sterol regulatory element-binding protein 1 (SREBF1), was downregulated in CTL JD(+) macrophages in comparison with CTL JD(–) macrophages (Supplementary Table 4). In contrast, some genes involved in fatty acid elongation (e.g., *ELOVL6* and *ELOVL7*) were upregulated in CTL JD(+) macrophages in comparison with JD(–) macrophages (Supplementary Table 4). A receptor of oxidized low-density lipoprotein (LDL), namely, *LOX1*, was upregulated in MAP-infected JD(–) macrophages during the 1-to-8-hpi period (Supplementary Table 4). In these CTL JD(+) macrophages, the gene expression of the LDL receptor, *LDLR*, was also increased while the *IDOL* (E3 ubiquitin ligase-inducible degrader of the LDL receptor) gene encoding the enzyme regulating its degradation was downregulated. While the expression of genes responsible for *de novo* lipid synthesis was reduced, LDL intake is supported by the increased expression of genes encoding LDL receptors or scavengers of modified LDL receptors and by increased expression of encoding fatty acid elongases in CTL JD(+) macrophages in comparison with CTL JD(–) macrophages.

#### Formation of Lipid Droplets and Macrophage-Derived Foam Cells

The KLF4 and c-MYC proteins are involved in macrophage polarization as well as in the formation of cytoplasmic lipid droplets (64). Both transcriptional activators *KLF4* and *MYC* were upregulated in CTL JD(+) macrophages vs. CTL JD(–) macrophages (Supplementary Table 4). Overexpression of these partners is sufficient to drive cell differentiation into adipocyte-like cells (65), which might influence polarization as well. The gene encoding the transcription factor early B cell factor 1 (*EBF1*), which regulates genes controlling lipolysis and adipocyte morphology and differentiation, was much more (>100-fold) abundant in CTL JD(+) macrophages than in CTL JD(–) macrophages (Supplementary Table 4). Other members of lipid oxidation and accumulation of lipid droplets were affected by JD status. The gene encoding perilipin 2 (*PLIN2*), which coats intracellular lipid storage droplets, was more ~2-fold abundant in CTL JD(+) macrophages than in JD(–) macrophages (Supplementary Table 4).

**Figure 3:**
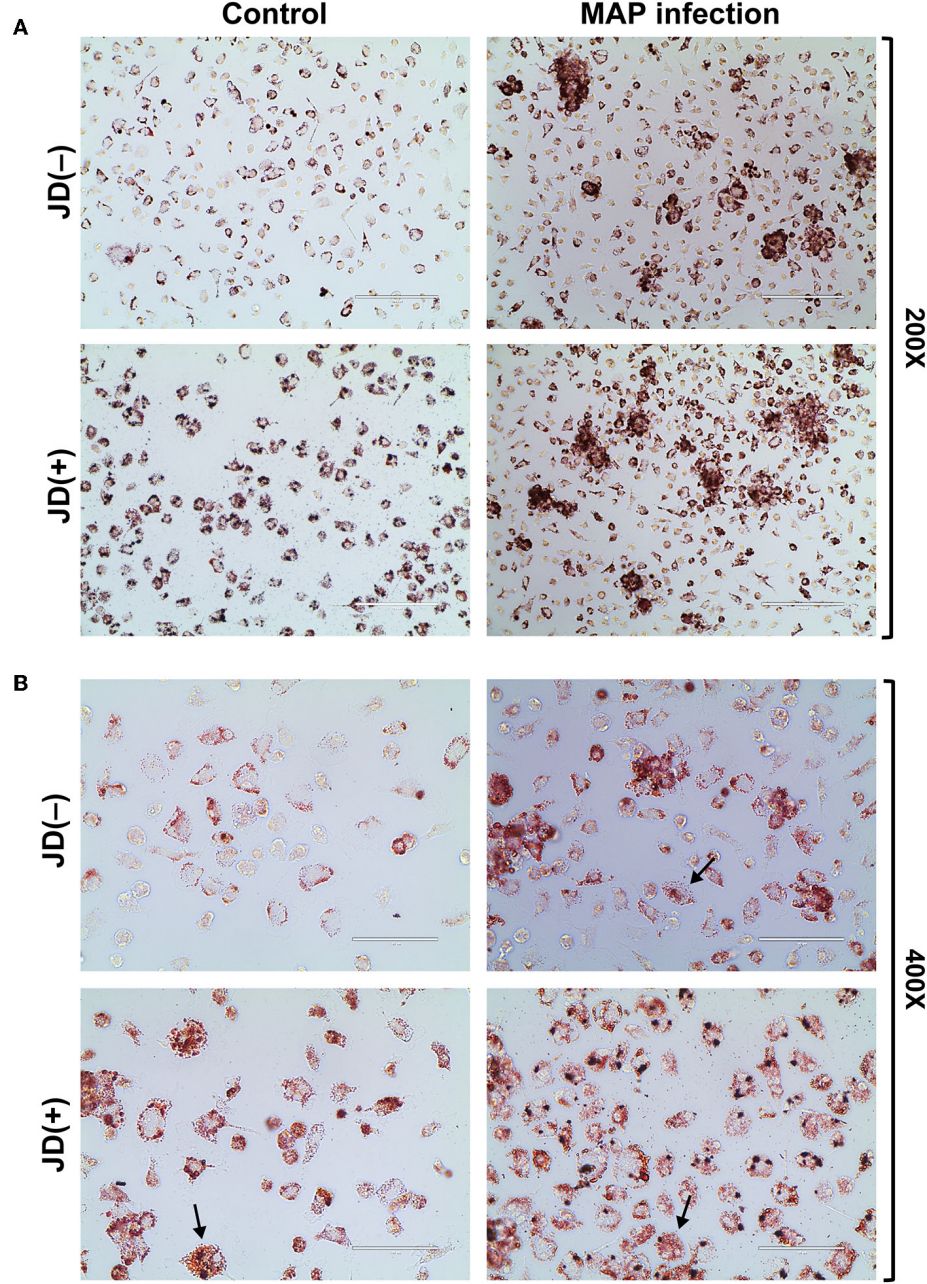
Lipid accumulation assay using Oil Red O staining. The staining is shown for macrophages from Johne's disease negative [JD(–)] and positive [JD(+)] cows and for uninfected (CTL) or MAP-infected (5 days) experimental condition. The images were observed at **(A)** 200× magnification (scale bar = 200 μm) and at **(B)** a 400× magnification (scale bar = 100 μm). Arrows show macrophages with a classical foam cell morphology suggesting macrophage-derived foam cells. These are representative images from three experiments. Lipid accumulation assay for macrophages from two additional JD(–) and JD(+) cows are given in Supplementary Figure 3.

As described above, the impact of MAP infection and JD status on several pathways pointed toward the accumulation of lipids in macrophages through the involvement of fatty acid metabolism and the cholesterol influx/exflux, among others. The accumulation of lipids in CTL and MAP-infected macrophages was monitored using an Oil Red O lipids staining assay. A representative result is shown in Figure 3. Microscopy analysis of CTL macrophages from JD(+) cows revealed staining levels higher than in CTL JD(–) macrophages despite the fact that these macrophages were not infected by MAP. Our results suggest that blood circulating monocytes from JD(+) cows differentiating into macrophages are already accumulating lipids without any apparent stimuli. The accumulation of lipids in MAP-infected macrophages from both JD(–) and JD(+) cows correlated with the presence of clumps of cells, as shown at the lower (200×) magnification (Figure 3A). Their granulomatous appearance suggests high endogenous lipid droplets, as clearly observed at the 400× magnification (Figure 3B), while very few lipid droplets were visible in unexposed JD(–) macrophages. The Oil Red O stain was seen mostly at the cytoplasmic membrane in these JD(–) CTL cells. This contrasted with MAP-infected macrophages, where lipid droplets accumulated throughout the cytosol of most cells. Interestingly, some macrophages from CTL JD(+) cows assumed the morphological appearance of foam cells with many Oil Red O lipid droplets, despite the fact that these cells were not infected by MAP (Figure 3B, left panel, arrows). Foam cells were also observed in both JD groups in response to infection (Figure 3B, arrows). These larger cells showed an abundance of lipid droplets that was grossly correlated with the morphological appearance of foam cells. This foamy cell phenotype would support the top Hepatic stellate cell (also known as fat-storing cell) pathway found in our enrichment analysis.

Overall, MAP had a major influence on both cholesterol metabolism and lipid homeostasis. Our results suggest positive lipid entry. In addition, the transcription factor PPARG and LXR/RXR-related pathways were negatively affected both in response to MAP infection and in CTL JD(+) macrophages. Finally, the accumulation of lipids in CTL JD(+) macrophages confirmed the lipid homeostasis shift that is linked to JD.

## Discussion

### Lipid Homeostasis

**Figure 4:**
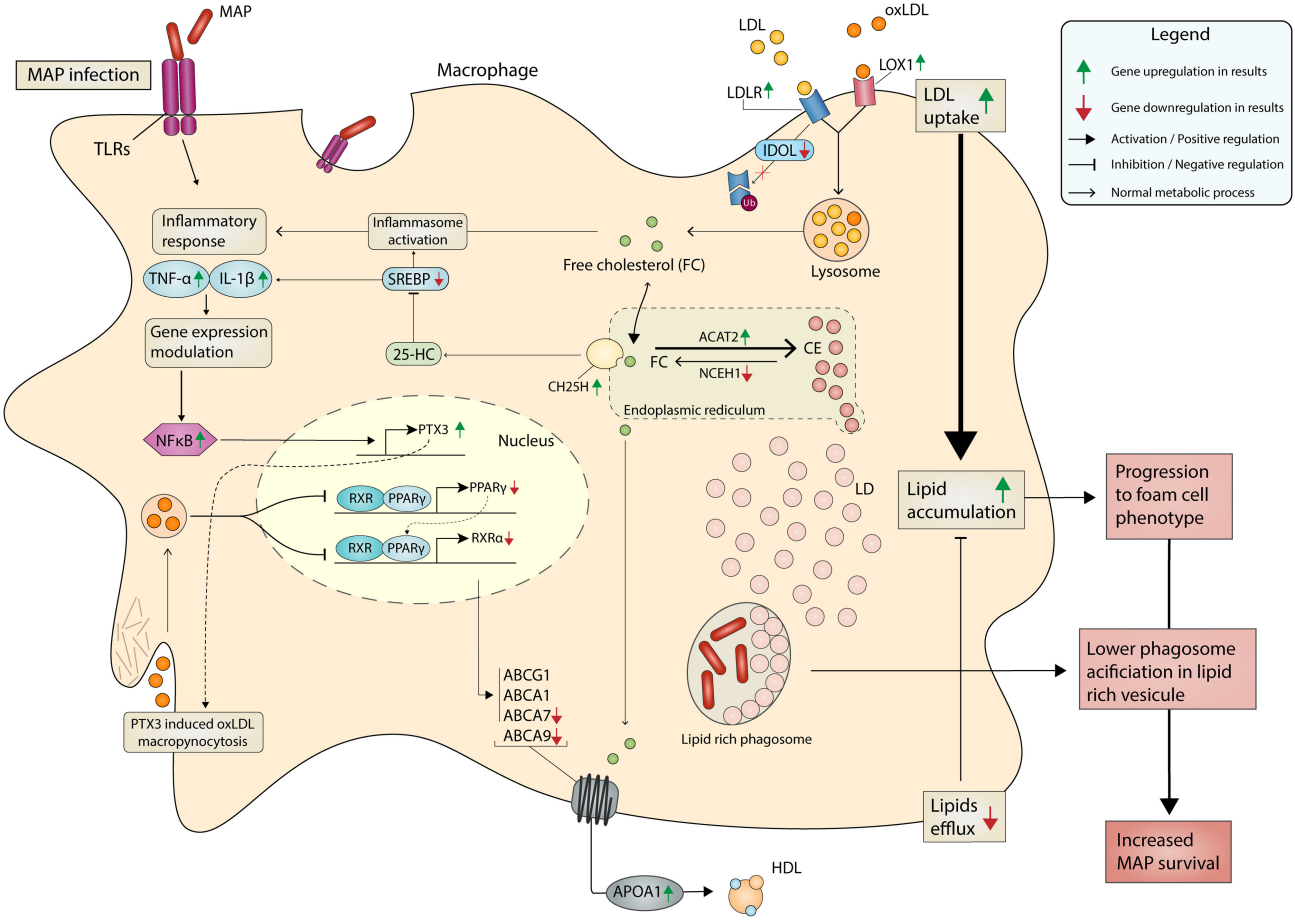
Metabolism in MAP-infected macrophage promotes lipid accumulation and chronic JD. MAP-mediated signaling induces an inflammation that results from the translocation of NF-κB (p50–p65 heterodimers) to gene promoter sites, as well as the assembly of a coactivator complex on these sites, together resulting in gene transcription, notably *IL1A, IL6, PTX3*, and *TNF* among others. In parallel, the lipid metabolism is greatly affected by MAP infection. Our results suggest that CTL JD(+) macrophages also depict a metabolism supporting lipid entry because scavenger receptors (LDLR, PTX, and SCAR5) were upregulated while the gene encoding LDLR degradation (IDOL) was downregulated. PTX3 promotes oxLDL uptake through macropynocytosis. The phagocytized LDL or oxLDL are released from the liposome as free cholesterol (FC) or oxLDL in the cell. Due to the higher expression of the *ACTA2* gene over *NCEH1* genes, which have reversed actions, the FC is then predominantly transformed in cholesterol ester (CE). These are then stored in the form of lipid droplets (LD). PPARs and LXRs are nuclear receptors activated by non-esterified fatty acids and cholesterol metabolites, respectively. The activation of PPAR-γ inhibits the expression of target genes through an epigenetic mechanism (histone deacetylase) and a process that prevents its degradation (inhibited ubiquitination) and thereby maintains the inflammatory genes in a repressed state. Sterol metabolites are natural ligands of LXRα, and LXRα is a sensor of cholesterol for controlling genes involved in a process known as reverse cholesterol transport. Downregulation of genes like *PPAR*γ and *LXR*α was observed in both MAP-infected and CTL JD(+) macrophages. They have a negative impact on the expression of cholesterol exporter genes like *ABCA7* and *ABCA9*, thus supporting the reverse cholesterol transport. The increased LDL uptake combined with limited lipid efflux induces lipid accumulation, which supports the phenotype of foam cell macrophages. It is well-known that phagosome acidification is limited in lipid-rich vesicles, which in turn supports MAP survival in macrophage. The DE genes observed by RNA-seq analysis are represented by upregulation and downregulation using green and red arrows, respectively.

At first glance, it makes sense that lipid droplets are an attractive target for MAP seeking energy resources from host macrophages. Lipid droplet (LD) formation occurs during activation of macrophages and also during infection of macrophages by intracellular pathogens, including *M. tuberculosis* (Mtb) (66). The HIF-1α-dependent signaling pathway implicated in host defense redistributes macrophage lipids into LDs (66). Interestingly, this *HIF1A* gene was DE in response to MAP infection (Table 2). The ability of Mtb to switch between glucose to host-derived fatty acids and cholesterol as sources of nitrogen, carbon, and energy during host infection is well-known (67). MAP is also able to use cholesterol as a carbon source (68) and manipulate host lipid metabolism during early infection (69). Our data support the fact that MAP promotes the uptake of LDL and modified LDL through the upregulation of some receptors (e.g., *LDLR, VDLR*, and *LOX1*). In addition, *MYLIP*, encoding the E3 ubiquitin ligase-inducible degrader of LDLR, was downregulated in macrophages of JD(+) cows compared to JD(–) macrophages. The reduction was even greater in response to MAP infection in JD(–) macrophages. These results suggest that reduction of *MYLIP*, encoding the LDLR ubiquitination enzyme IDOL, might promote a sustained cell-surface LDLR level with the consequence of lipid accumulation observed in JD(+) macrophages (Figure 3), as schematized in Figure 4.

Accumulation of lipids can also be induced by PTX3 (53), which is upregulated by TLR agonists such as the Mtb cell component lipoarabinomannan (70). In bovine macrophages, PTX3 was greatly upregulated during MAP infection, which confirms these former results and suggests that manosylated lipoarabinomannan from MAP has the potential to upregulate *PTX3*. The constitutive expression of *PTX3* is allowed by transcription binding factors, notably AP-1 and SP1, whose binding sites are located in its promoter (71). In addition, it contains the NFκB binding site and thus can be induced by several primary inflammatory signals, including TNFα and microbial components (72). In the CTL macrophages from the JD(+) cows, *PTX3* was upregulated 26.7-fold (Table 2) and lipids accumulated at a greater level in these cells (Figure 3) even though they were not infected. Studies on colorectal cancer reported evidence that methylation at the *PTX3* promoter affects plasmatic PTX3 levels (73). For JD, an epigenetic modification at the *PTX3* promoter could be one mechanism to explain the higher *PTX3* expression in CTL JD(+) macrophages.

The role and action of PTX3 is not restricted in the defense against microbial infections. Overexpression of PTX3 in human macrophage using a recombinant gene increased total lipid content and reduced cholesterol efflux (53). The association of foamy phenotype and colocalization of PTX3 was described in advanced human atherosclerotic plaques (74, 75). We hypothesize that upregulated *PTX3* can also contribute to the development of foam cell phenotype observed in MAP-infected or JD(+) macrophages (Figure 3) as schematized in Figure 4. Monocyte/macrophages play a pivotal role in the initiation and progression of atherosclerosis. The accumulation of lipid, the reduction of cholesterol efflux, and the expression of *PTX3* are particularly interesting in the context of mycobacterial infection. Notably, the presence of antibodies against mycobacterial or lipoarabinomannan cell wall antigen has been found in patients with atherosclerosis (76) and rheumatoid arthritis (77, 78). Our pathway analysis revealed an association of MAP infection with these chronic diseases through “Role of macrophages in rheumatoid arthritis” (Supplementary Figure 4) and “Hepatic fibrosis and Atherosclerosis signaling pathways” (Supplementary Figure 5). Although *PTX3* is not yet included in IPA pathways, the literature and our results suggest a potential role for PTX3 in them. Moreover, PTX3 should also be included in the classic PPARγ-LXRα-ABC transporter pathway, which pumps cholesterol out of macrophages. Our data corroborate observations reporting that PTX3 downregulates *PPARG, NR1H3* (LXRα), and LDL transporters (*ABCA7* and *ABCA9*) (53).

LXR/RXR is active in heterodimer complexes and controls many genes that regulate lipid uptake/efflux (79). MAP-infected macrophages downregulated both *PPARG* gene and the RXR encoding gene *NR1H4*, which in turn negatively affected the expression of their targets, namely, the cholesterol efflux transporters *ABCA7* and *ABCG9* genes. In addition, *ABCA7* and *ABCA9* were lowly expressed in the CTL JD(+) macrophages compared to the level observed in CTL JD(–) macrophages. The ABC lipid transporters are involved in the movement of cholesterol, lipid homeostasis, and inflammation (80). The reduction of RXR limits the cholesterol efflux in a process termed reverse cholesterol transport. The downregulation of *ABCA7, ABCA9*, and *NR1H4*, together with the increased expression of *PTX3* and the lipoproteins that traffic modified cholesterol and triglycerides through members of the LDL receptor family, notably *LDLR, MYLIP, VLDLR*, and *LOX1*, supports sterol deposits in macrophages as suggested in Figure 4. Interestingly, genes related to the LXR/RXR and PPAR pathways are also among the DE genes in foam cell macrophages (81). We suggest that increased *PTX3* expression, which has a negative impact on *PPARG* and *NR1H4* (RXR) expression, increases lipid uptake including oxidized LDL, and reverses cholesterol transport, and thus supports the foamy phenotype of JD(+) macrophages (Figure 3).

Re-esterification is a protective mechanism to prevent the toxicity of excess free cholesterol in cell membranes (82). Our results suggest that the sustained *ACAT2* expression over *NCEH1* observed during MAP infection and in CTL macrophages from JD(+) cows would be protective for the host as schematized in Figure 4. Interesting hypothesis can be drawn from our results and supported by observations from a study on the effect of cholesterol loading on macrophage foam cell lysosomes. Cox et al. reported that cholesterol trafficking toward intracellular membranes, notably to lysosomal compartments, contributes to the loss of acidity that impaired their function (83). On one side, host acetylates cholesterol to reduce its toxicity, but on the other hand, MAP may upregulate genes encoding cholesterol synthesis proteins (e.g., *CYP51A1, CD38*, and *HMGCR*) for a protective effect on cholesterol loaded lysosomal membranes. This finding is particularly interesting considering that MAP localizes to cholesterol-rich compartments in macrophages (18) and that cholesterol impairs phagosome maturation (84). By further increasing intramembranous cholesterol levels, MAP could sabotage the protective role of lysosomes, while lipid droplets accumulate and predispose MAP survival by providing carbon-rich energy. Lipid metabolism is at the host:pathogenic mycobacteria interface. The importance of lipids derived from the host in mycobacterial pathogenesis has been identified for Mtb (85). Our results and the relevant literature on other mycobacterial diseases provide support for the role of host lipid accumulation in the pathogenicity of MAP. Owing to the sensitivity of RNA-seq analysis, a model of potential pathogen–host interactions involving these novel pathways was developed and suggests an important role for host lipids in MAP survival and persistence.

### M1/M2/Mreg Polarization

Understanding the impact of JD on the immune system of the ruminants remains a challenge. MAP belongs to the family *Mycobacteriaceae* and even if classified under the non-tuberculous bacilli, MAP induces a disease with many pathogenic features common to tuberculosis. Clearance of Mtb by the host promptly tilts macrophages toward M1 polarization which was also observed in MAP-infected primary JD(–) macrophages using the M1 fluorescent cell marker CD80 (data not shown). Moreover, in Mtb infection, the positive feedback loop of *NOS2* involves the transcription factor HIF-1α. In macrophage, HIF-1α coordinates an immunometabolic shift to aerobic glycolysis essential for controlling Mtb infection (86, 87). A similar transcriptomic trend was observed for MAP-infected macrophages in this study (Table 2). MAP-infected JD(–) macrophages might have to rely on rapid glycolysis for their ATP production, which is amplified by HIF1α (86), thus responding to the need for energy and for a rapid immune response. In our study, numerous genes associated with classically activated or M1 macrophages were found significantly upregulated, notably *HIFA, SLC2A1, SLC2A3*, and *SLC2A6*. At the same time, *CARKL*, which is associated to M1 polarization, was repressed by MAP infection. Downregulation of *CARKL* is critical for driving the macrophage metabolism toward increased pentose phosphate pathway activity and increased redox state, for supporting M1 polarization (46). Together, our data suggest that a clear metabolic switch occurs during the early event of MAP infection, suggesting a proper metabolic reprogramming as described previously (45).

Resolution of Mtb inflammation has been reported when Mtb entered dormancy. The phenomenon concurs with M2 polarization with an increased production of arginase and anti-inflammatory cytokine such as IL-10 (24). While *ARG1* was not affected in our study, *ARG2* and *IL10* promptly increased and their level remained elevated at 24 hpi. Arginine bioavailability is a critical element to NOS2 activity and its limitation contributes to reduce nitric oxide synthesis in mitochondria (88). For some intracellular pathogen, notably *Helicobacter pylori*, ARG2 supports immune evasion by controlling M1 macrophage activation (89). Limiting arginine availability to NOS2 as early as 1 hpi is a mechanism that MAP may use to promptly reduce the oxidative stress and support its survival. It is noteworthy that ARG2-positive macrophages prevailed in chronic inflammatory lesions of atherosclerosis patients (90). In our results, the “Atherosclerosis Signaling” was among the significant pathways at 1 hpi and still ranked second at 24 hpi in response to MAP in JD(–) macrophages. While the host might want to shape macrophages in a pro-inflammatory M1 polarized status, it is noteworthy several M2 markers were also detected, notably *EDN1* and *KLF4*. Like Mtb, MAP might exploit macrophage signaling pathways to orchestrate a response that dictates a bias toward M2 polarization on the long run. An example is Mtb using KLF4 to tilt macrophage response toward the production of arginase and the inhibition of autophagy. KLF4 inhibits M1 and activates M2 polarization (91) while the EDN1 factor mainly exerts a pro-fibrotic effect (92). In MAP-infected JD(–) macrophages, *KLF4* and *EDN1* were found strikingly induced. In addition to the transcriptional activator KLF4, c-Myc is also involved in the formation of cytoplasmic lipid droplets (64). The activity of PRMT1 is necessary for c-Myc transcriptional function in M2 polarization (48) and they were increased in MAP-infected JD(–) macrophages. Altogether, their effects associated with M2 polarization and lipid accumulation also support a role in the development of the foamy shape observed in response to MAP infection (Figure 3).

In CTL JD(+) macrophages, the higher levels of *IL10, KLF4, MYC*, and *EDN1* compared to CTL JD(–) macrophages also support the foamy phenotype observed in these cells (Figure 3). It suggests a priori M2 phenotype in the JD(+) CTL cells, even though its content of the M1 marker, CD80, was more abundant compared to JD(–) macrophages. Similar observations were reported for experimentally MAP-infected cattle (20). They observed that MAP infection did not lead to a specific polarized pattern of the circulating monocytes. Although our main objective was not to characterize the dominant polarization phenotype, the mixed surface markers or inducible effector molecules observed in JD(+) macrophages might suggest a mixed M1/M2 polarization phenotypes in JD(+) macrophages. Indeed, the dichotomy M1/M2 has been reconsidered (20). And now, the regulatory M3 is described as a mixed M1/M2 macrophage phenotype (93–95). This subset can induce Th17 polarization (89, 95). It is particularly noteworthy that we found IL17 more elevated in the blood of JD(+) cows in a previous study (11). In addition, we found *CCL20* among the most highly upregulated genes in MAP-infected JD(–) macrophages, which also remained higher in CTL JD(+) macrophages (Supplementary Table 4). This cytokine is known to induce a strong chemotactic response and it is involved in the recruitment of both Th17 and Treg cells to sites of inflammation (96). Globally, our results suggest that blood circulating monocytes from JD(+) cows can potentially differentiate into macrophages under a mixed M1/M2 macrophage phenotype and accumulate lipids, while predisposing MAP survival by providing rich carbon source energy.

### Resolution of Inflammation

**Figure 5:**
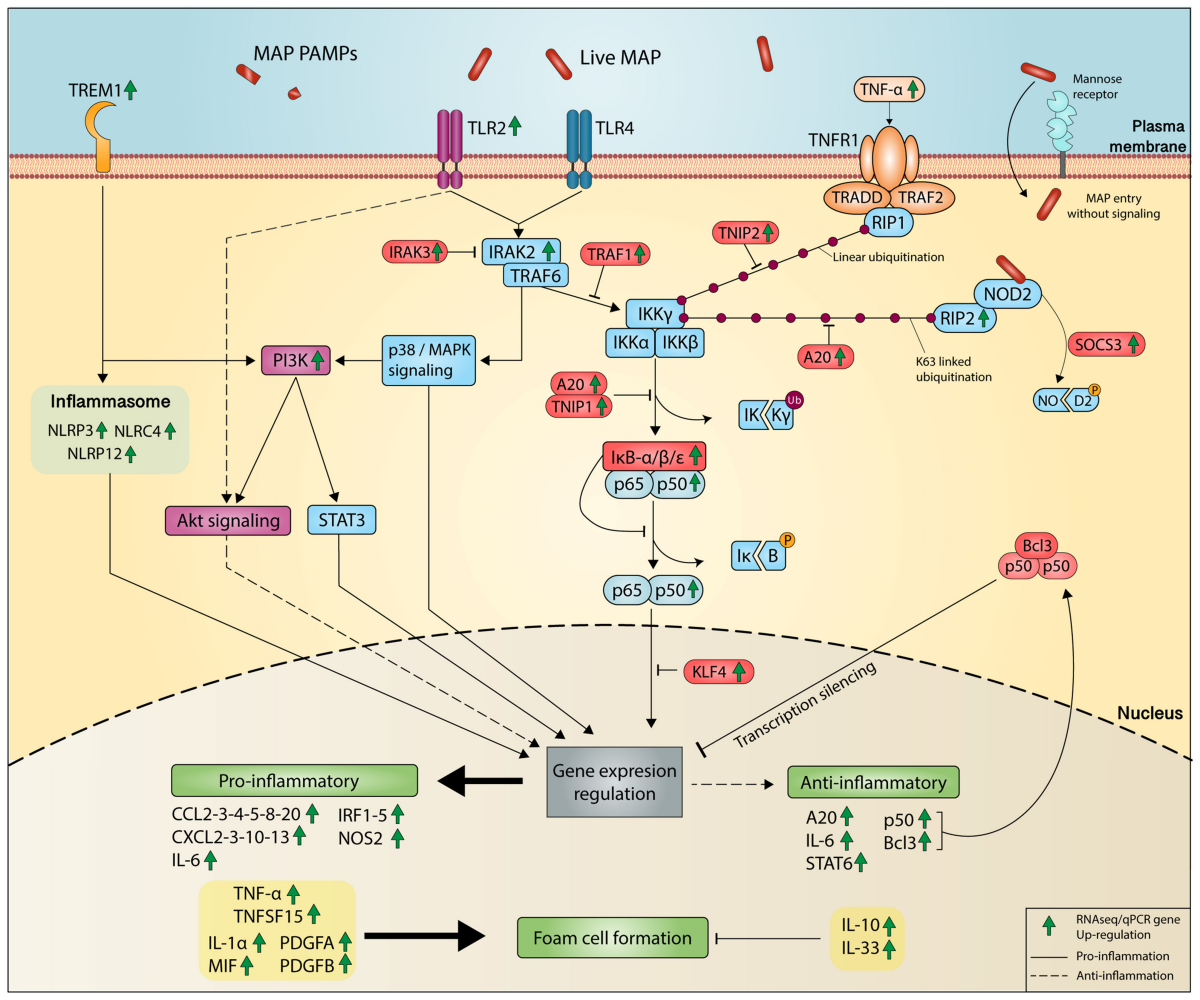
Molecular evidence of inflammatory response and resolution of inflammation during MAP infection. In the context of infection, the presence of MAP and/or its associated PAMP in the extracellular space is recognized at the plasma membrane of macrophage by TLR2, TLR4, TREM1, and mannose receptors. The TLRs and mannose receptor induce the MAP phagocytosis. The activation of canonical NF-κB pathway depends on the IKK complex, which contains two catalytic subunits (IKKα and IKKβ) and a regulatory subunit/IKBKγ (alias NEMO). Ubiquitination of IKBKγ activates these two associated kinase enzymes. Catalytically active IKKβ phosphorylates IκBα, signaling its ubiquitination toward its proteasomal degradation allowing the p65/p50 NF-kB heterodimer to translocate into the nucleus and to bind specific recognition sequences in genes (referred to as kB sites) to modulate transcription. Tight control of the IKK complex fate is controlled by numerous members. Members of the pro-inflammatory NFκB pathway (represented in blue) are activated by the TLR signaling with the help of IRAK proteins like IRAK2. Many genes that encode inhibitor (represented in red) of the NFκB pathway were upregulated and contribute to the resolution of the inflammation by avoiding overactivation of the NFκB pathway. Detection of intracellular MAP by the complex NOD2-RIP2 contributes to release NOD2. In turn, RIP2 contributes to the ubiquitination of IKKγ and SOCS3 induced by NOD2 activation, which further facilitates its degradation process and thus limiting NOD2 sensing capability. As *TNF* was greatly upregulated during infection, TNFα might activate TNF pathway that in turn supports linear ubiquitination between RIP1 and IKKγ, which are regulated by the inhibitor TNIP2. IRAK3 is a downstream negative effector of TLR signaling. The TLR activation triggers the p38/MAPK signaling that impacts the PI3K activation of anti-inflammatory (represented in purple) AKT signaling and STAT3 signaling. TREM1 pathway is involved in the innate inflammatory response to microbial infections and amplifies the TLR-mediated inflammation signaling pathway. In addition, TREM1 pathway induced the activation of the NOD-like receptor (NLR) inflammasome. While the translocation of p65/p50 NF-kB heterodimer specifically upregulated NFκB target genes, overexpression of transcription silencers p50 homodimer stabilized in the Bcl3 complex negatively controls NFκB target genes. Several genes associated with the formation of foam cells were upregulated and may support the foamy phenotype observed in MAP-infected macrophages. The significant upregulation observed in RNA-seq of the genes associated to the proteins represented is indicated by a green arrow.

Similar to Mtb, the recognition of MAP is mediated through various pattern recognition receptors, including but not limited to TLRs, C-type lectin receptors, mannose receptors, and NOD-like receptors (97, 98). Globally, our RNA-seq analysis of the signaling cascades supports the recognition of these receptors, including TREM1 signaling (Figure 5). Several NLR family members downstream of the “TREM1 signaling” pathway were upregulated, notably *NLRP3–4* and *NLRP12*. Whereas, the ligand for TREM1 is unknown, TREM1 is considered a potent amplifier of the inflammatory response to invading microbes (99). Whether MAP is recognized by TREM1 receptor is not yet known. However, TREM1 may act as a key player in protective innate immunity during MAP infection. While *NOD2* was not DE, *SOCS3* was greatly upregulated upon MAP infection. In response to MAP infection, the protein SOCS3 would degrade NOD2, limiting its sensing capability (Figure 5). In MAP-infected macrophages, downstream of the TLR-induced pathway, *IRAK2, IRAK3*, and *TRAF1* were upregulated (Figure 5). Enhanced *TRAF1* gene expression and protein levels have been reported for infected bovine macrophages (100) and ileal tissues form JD cows (101). *TRAF1* suppresses the alternative NF-κB pathway, thus prioritizing the classical NF-κB pathway (102). Upregulation of *TRAF1* is particularly interesting for MAP persistence as TRAF1 promotes immune cell survival (103).

Supported by the rapid upregulation of *TNF* and sustained expression of some associated receptors (e.g., *TNFRSF12A, TNFRSF18, TNFRSF1B*, and *TNFRSF6B*), the NF-κB activating pathway might be highly solicited. This is confirmed by the upregulation of several NF-κB target genes identified DE in MAP-infected JD(–) macrophage. Overall, among the 359 genes regulated by NF-κB identified in the RNA-seq analysis, 174 (34%) were identified DE at least at one time point of the MAP infection in JD(–) macrophages (data not shown). The top DE genes with the highest upregulated fold changes include many interleukins (*IL6, IL1A*), chemokines (*CCL8, CCL4, CCL20*), and colony-stimulating factors (*CSF2, CSF3*). Moreover, pathways such as “IL-10 signaling,” “IL-6 signaling,” “NF-kB signaling,” “Acute phase response signaling,” and “Differential regulation of cytokine production in macrophages and T helper cells by IL-17A and IL-17F” showed strong regulation of immunity-related genes during early infection. Our results for MAP-infected JD(–) macrophages confirmed previous findings obtained by Casey et al. at 2 hpi but drastically differed from the 6-hpi time point in the same study (21). Notably, the “IL-10 signaling” pathway ranked 149th at 6 hpi in that study, whereas, in ours, we observed a more consistent and continuous progression of the infection in terms of the number and composition of DE genes and their associated pathways. Our study is in agreement with another study identifying “IL-10 signaling” as a canonical pathway targeted by MAP to control the immune response (31). We believe that our results adequately reflect the initial events involving live and active MAP during the initial 24-h infection period. By mobilizing immunity-related genes, the host attempts to control the invading pathogen; however, MAP sustains the “IL-10 signaling” pathway, indicating that it also has an immunosuppressive effect. We can advance a plausible explanation for the difference observed in a previous study where IL-10 signaling was no longer dominant beyond 6 hpi, namely, that frozen aliquots of MAP were used in divergent studies for *in vitro* infection (21, 104), as opposed to the live MAP used in our study. The discrepancy between the 6-hpi time point found in these studies and in our study may be attributable to the fact that macrophages are better at removing dormant MAP pathogens. The importance of using live mycobacteria was also highlighted in a previous Mtb study (105). CORO1A has been considered as an essential host protein that allows the intracellular survival of live mycobacteria in macrophages (106). Coronin 1 is retained on phagosomes containing viable, but not killed mycobacteria and its retention on phagosomes prevents its fusion to lysosome and intracellular killing of live Mtb (105). In our study, *CORO1A* was upregulated in macrophages in response to MAP infection. CORO1A was not identified as DE in a previous study using non-live MAP in macrophage infection (21) but was DE *in vivo* in JD cows (107).

Several pathogens use more than one strategy to circumvent host cell destruction; in fact, Mtb is one of those most notorious pathogens and MAP seems to also deploy multiple arsenal to protect its niche (98). As mentioned above, and given the observed high expression of *TNF*, the TNFα receptor-mediated pathway can eventually deviate from the NF-kB pathway toward a programmed cell death. However, primary bovine macrophages infected with MAP are less likely to undergo apoptosis than control, uninfected cells (108). Apoptosis can be compromised because of TNIP1, which prevents the recruitment of procaspase 8 and the complex can no longer support apoptosis (109). Our results suggest that MAP may prevent apoptosis by upregulating *TNIP1* and its protein–protein partner *TNFAIP3* (A20). This mechanism is known to synergistically impair procaspase 8 recruitment (110). The reduction of caspase 8 activity was observed in primary bovine macrophages infected by MAP (108). In addition to compromising apoptosis, several other mechanisms are needed to refine the inflammation to limit damage to the host. Those two important modulators, namely, TNIP1 and TNFAIP3 (A20), also cooperate to restrict NF-κB activation, i.e., for the resolution of inflammation. They form a paralyzing complex with the essential regulatory subunit IKKγ (also known as NEMO) that regulates NF-κB activation (55). In MAP-infected macrophages, both *TNIP1* and *TNFAIP3* (A20) were upregulated and might prevent the activation of the IKK kinases (Figure 5). In addition to *TNIP1*, a second inhibitor, namely, *TNIP2*, was upregulated. TNIP2 significantly inhibit NF-κB activation as it prevents the interaction of IKKγ/NEMO with RIP1, which impairs IKKγ/NEMO ubiquitination (111). Its ubiquitination is required for the activation of the IKK kinases from the complex. In MAP-infected JD(–) macrophages, TNIP2 would impair IKKγ/NEMO interaction with IKK kinases and thus restricts NF-κB activation. A priori upregulation of both *NOD1* and *RIPK2* in MAP-infected macrophages would suggest an efficient host control over the intracellular infection. Indeed, interaction of NOD1 and RIP2 kinase (RIPK2) induce the ubiquitination of NEMO (112). However, TNFAIP3 (A20) prevents NF-κB activation by inactivating RIPK2 (113, 114). Altogether, these results suggest that MAP promptly supports resistance to apoptosis and that numerous control points dampen NF-κB dominant activity at the early stage of the infection, thus supporting both MAP and host survival.

### Immune Tolerance in Perspective to Explain Refractory State of JD(+) Macrophages

If it cannot eliminate the pathogen, the phagocyte must adapt to its imposed resident. For JD, it becomes apparent that host–MAP coexistence is not simply a lack of TLR or Th1-cell responsiveness but rather an adaptation of the innate immune response to persistent exposure to MAP components. At first glance, our RNA-seq data suggest a host–pathogen duality promoting lipid accumulation under the signature of M1/M2 characteristics. However, some additional molecular mechanisms pointed toward a long-term programming profile of immune-tolerance. *IRAK3* (alias IRAK-M), whose expression is restricted to macrophages, is required for endotoxin tolerance (115). Whether IRAK3 is required for inducing MAP tolerance through the TLR pathway as schematized in Figure 5 requires further investigation. Beyond both TLR- and TNF-signaling pathways during MAP infection, the classical NFκβ negative feedback system, as schematized in Figure 5, can also induce tolerance. While the endotoxin tolerance is generally mediated by TLR4, other microbial products can stimulate receptors such as TLR2 (116) or TNF receptor (117) to induce cross-tolerance. TNF selectively diminished cytokine production in response to subsequent endotoxin challenge (118). A low dose of TNF protected mice from the lethal effects of subsequent challenge with a high dose of LPS (117). In CTL JD(+) macrophages, *TNF* upregulation was maintained compared to JD(–) CTLs. Genes that are critical for the induction of tolerance in human monocytes such as *TNF* and *TNFAIP3* (encoding A20 protein) (118, 119) were also more expressed in control JD(+) macrophages than in CTL JD(–) macrophages. Our results suggest a phenomenon similar to the cross-tolerance in these JD(+) macrophages through a mechanism that requires further investigation. Although the molecular basis of dampened response in JD(+) macrophages has remained elusive, we hypothesize that MAP establishes a form of innate immunologic memory. If confirmed, the mechanism that MAP established in its phagocyte is called tolerization of inflammatory gene expression (120). This transcriptional tolerization (or cross-tolerization) of proinflammatory genes during systemic inflammation involves an epigenetic process that disrupts NF-κB transactivation (121, 122).

**Figure 6:**
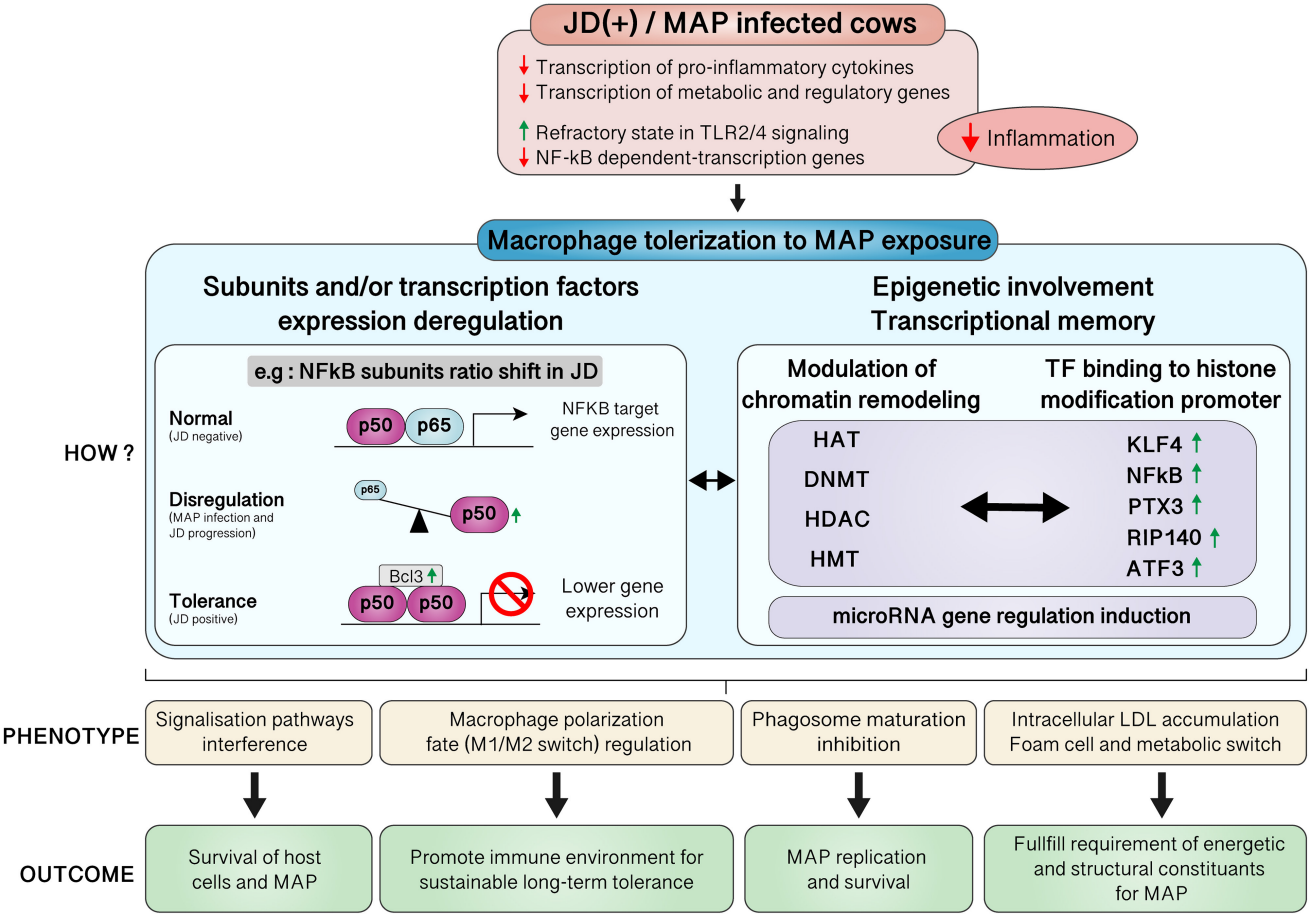
Mechanism of tolerance induced by MAP in bovine macrophages. The mechanisms that support a long-term refractory state of JD(+) macrophages that dampens their pro-inflammatory response to subsequent stimulation are still unknown. While the promoter κB-binding motif-dependent genes and regulatory genes of the NFκB pathway were DE in JD(–) macrophages in response to a first exposure to MAP, they showed a refractory inflammation state in JD(+) macrophages. In addition, the shift of NFκB subunit ratio toward the overrepresentation of the p50 subunit was observed. The role of homodimer p50 in tolerance has been described (123, 124), which is further supported by the increased expression of *BCL3* that stabilized the homodimer that in turn inhibits the transcriptional activity of NF-κB (125). The NFκB regulated genes are also the targets for epigenetic programmers. After rapid initiation of the acute systemic inflammatory process, gene-specific epigenetic reprogramming shifts the TLR-NF-κB-p65-dependent initiation phase to the adaptation phase (126). Altogether, they regulate epigenetic silencing during endotoxin tolerance (127). Our data support the fact that the expression of putative tolerized genes was promptly induced in MAP-infected JD(–) macrophages but limited in JD(+) macrophages, suggesting a long-term refractory state similar to the endotoxin tolerance. Those epigenetic modifications might also influence the functional capacity of the macrophages by altering the expression of gene associated with macrophage polarization, phagosome maturation, and the intracellular accumulation of lipids associated to the foam cell phenotype.

From our data, the shift in the NF-κB subunit ratio toward the overrepresentation of p50 (*NFKB1*) during MAP infection (Figure 5) supports this hypothesis of epigenetic transcription silencing. The role of the p50 homodimer in tolerance has been previously described (123, 124). Our data thus suggest a recruitment of NF-kB complexes composed mainly of p50/p50 homodimers. This complex cannot induce transcriptional activity but rather recruit and increase methylation of histones (120). Furthermore, the increased expression of *BCL3*, which stabilizes the homodimer (125), suggest that p50/p50 homodimer in turn inhibits the transcriptional activity of NF-κB (Figure 6). Our data suggest that after rapid initiation of the acute systemic inflammatory process, gene-specific epigenetic reprogrammers shift the TLR-NF-κB-p65-dependent initiation phase to the tolerization phase by involving p50, ATF3, and A20 among others, as described in another study (126). Indeed, several genes having a function in epigenetic programming were found DE in MAP-infected macrophages. For instance, ATF3 is known to bind and repress not only its own promoter but also other ATF3 target genes through an epigenetics mechanism (128). The sustained *ATF3* expression throughout the MAP infection period suggests that ATF3 repressive regulation may suppress proinflammatory chemokine production, notably through, interestingly, NOD2-activated gene expression (129). LOXL2 specifically deaminates trimethylated H3K4, resulting in the generation of non-methylated H3K4 (58), thus moving from a transcriptional active state in MAP-infected JD(–) macrophages to the repressive methylation state in JD(+) macrophage (130). It is noteworthy that *LOXL2* remained 10 times more expressed in JD(+) macrophages compared to JD(–) CTLs. The HDACs and reduction of deamination activity at NF-κB target genes represent an unconventional and sparsely characterized way of epigenetic gene regulation in response to MAP infection. Shaping the NF-kB transcriptional response to MAP infection at NF-κB-regulated gene promoters would be the function of the epigenetic programmers (131). *DNMT1*, which was DE at an advanced stage in MAP-infected JD(–) macrophages, is necessary to establish and maintain DNA methylation patterns (132). *GADD45A* was strongly downregulated and therefore probably does not favor active DNA demethylation (57). Together, these results suggest the preservation of DNA methylation patterns at the sequence level.

Overall, we have identified DE genes encoding epigenetic programmers affecting DNA methylation and a second group of DE genes attributable to the chromosomal packaging activity. In addition, it is possible to consider that post-transcriptional molecular mechanisms [for example, microRNAs and ncRNAs (130)] would affect the memory of macrophages in JD cows. Our study did not aim to study the phenomenon of tolerance but our results suggest that these phenomena may affect MAP survival in the long run. The attenuated immune response of JD (+) macrophages might be embodied by epigenetic modifications (Figure 6).

## Conclusion

In conclusion, our study highlights potential functional roles for genes that have not previously been implicated in the host response to infection with MAP bacilli. While some classical pro-inflammatory genes were found to enrich immune pathways, others were associated with inflammatory resolution or chronic disease. Interestingly, lipid homeostasis and metabolism pathways were already affected in macrophages from cows with a long-standing JD infection. The present study describes a deep transcriptomic survey of uninfected JD(+)-exposed macrophages, which suggests that a foam cell phenotype participates in chronic JD inflammation. Finally, our results suggest that blood-circulating monocytes from JD(+) cows were already in a recalcitrant state similar to immune tolerance-type phenotype. Our findings raise many questions and highlight areas of opportunity for future research on this disease. The functional impact of the gene expression changes occurring at the protein and phenotypic levels should be investigated more thoroughly in follow-up studies. In order to identify the nature of the non-specific effects of paratuberculosis on macrophages, researchers now clearly need to consider epigenetic programming and immune tolerance in their future investigations.

## Data Availability Statement

The datasets generated for this study can be found in the RNA-seq data have been deposited in the NCBI Gene Expression Omnibus (GEO) database under the accession number GSE98363 (https://www.ncbi.nlm.nih.gov/geo/query/acc.cgi?acc=GSE98363).

## Ethics Statement

The animal study was reviewed and approved by Commité Institutionnel de Protection des Animaux du Centre de Recherche et de Développement de Sherbrooke d'Agriculture et Agroalimentaire Canada (ethical approval protocols 424 and 431). Written informed consent was obtained from the owners for the participation of their animals in this study.

## Author Contributions

NB conceptualized the study, obtained funding, and supervised graduated students. P-LD and OA performed the cell culture. OA conduced bioinformatics analysis. DG and OA performed qPCR for validation. NB, DG, and OA analyzed the data. NB and OA wrote the manuscript. EI-A and NG provided inputs in interpretation of the results. All authors contributed to the interpretation of the results and approved the final manuscript. NB was the principal investigator of the project.

### Conflict of Interest

The authors declare that the research was conducted in the absence of any commercial or financial relationships that could be construed as a potential conflict of interest.

## Supplementary Material

Supplementary Figure 1 Experimental design. Monocytes were isolated from Johne's disease negative [JD(–)] and positive [JD(+)] cows and cultured *in vitro* to support their differentiation into macrophages before the infection with live MAP at a MOI of 10:1. The 4 and 24 h controls (CTL), which are surrounded with a dotted line, were used as biological replicates for the RNA-seq and qPCR analyses.

Supplementary Figure 2 Evaluation of MAP uptake by macrophages from Johne's disease negative **(A)** and Johne's disease positive **(B)** cows. The infection efficiency was evaluated *in vitro* using fluorescence microscopy after 8 and 24 h incubation with MAP. These images, which are representative of three independent experiments on three different cows, show control (CTL) uninfected cells and 8 or 24 hpi of MAP infected macrophages. The images were merged from an observation of stained mycobacteria shown in green (fluorescent Auramine-O staining), nucleic acid stained in blue (DAPI), and differential interference contrast at 630× magnification. Scale bar = 40 μm.

Supplementary Figure 3 Lipid accumulation assay using Oil Red O staining for two additional cows for uninfected (control) or MAP-infected (5 days) experimental condition. The images were taken at **(A)** 200× magnification (scale bar = 200 μm) and at **(B)** a 400× magnification (scale bar = 100 μm). These are representative images from three experiments.

Supplementary Figure 4 Role of macrophages, fibroblasts and endothelial cells in rheumatoid arthritis pathway. This IPA pathway is the second most enriched by significant DE genes at 8hpi of JD(–) macrophages challenged with MAP. Shades of green represent the upregulated fold-change values of RNA-seq data and shades of red represent the downregulated values from MAP infected macrophages.

Supplementary Figure 5 Early signaling events in fat storing cells (also known as stellate cells). The IPA top-ranked “Hepatic fibrosis/hepatic stellate cell activation” pathway in primary MAP-infected macrophages: **(A)** at 8 hpi of JD(–) macrophages compared to CTL JD(–) and **(B)** for the comparison of CTL samples of JD(+) vs. JD(–) macrophages. Shades of green represent upregulated significant DE genes and shades of red represent the downregulated significant DE genes.

Supplementary Figure 6 “LXR/RXR activation” pathway significantly enriched by significant DE genes of JD(+) CTL macrophages. Shades of green represent upregulated genes compared to JD(–) macrophages, while shades of red represent downregulated genes as reported by the RNA-seq analysis.

Supplementary Table 1 Primer sequences used for qPCR validation.

Supplementary Table 2 Efficiency of *in vitro* infection of macrophages exposed to live MAP^1^.

Supplementary Table 3 Metrics of the RNA-seq data from 72 libraries of MDM from both JD status (± SD).

Supplementary Table 4 Gene expression (fold-change) and statistics (q-values) of genes at all time points.

Supplementary Table 5 Top 30 IPA pathways enriched by DE genes at 1 hpi in macrophages from JD (–) cows.

Supplementary Table 6 Top 30 IPA pathways enriched by DE genes at 4 hpi in macrophages from JD (–) cows.

Supplementary Table 7 Top 30 IPA pathways enriched by DE genes at 8 hpi in macrophages from JD (–) cows.

Supplementary Table 8 Top 30 IPA pathways enriched by DE genes at 24 hpi in macrophages from JD (–) cows.

Supplementary Table 9 Top 30 IPA pathways enriched by DE genes for the comparison of CTL macrophages: JD(+) CTL macrophages vs. JD(–) CTL macrophages.

Supplementary Table 10 IPA pathways at 4 hpi over-represented by DE genes in macrophages from JD (+) cows.
