# Supplementary figures and images for: Transcriptome Profiling of Bovine Macrophages Infected by Mycobacterium avium spp. paratuberculosis Depicts Foam Cell and Innate Immune Tolerance Phenotypes
Source: Front Immunol. 2020 Jan 8;10:2874. doi: 10.3389/fimmu.2019.02874 (PMC6960179; doi:10.3389/fimmu.2019.02874)

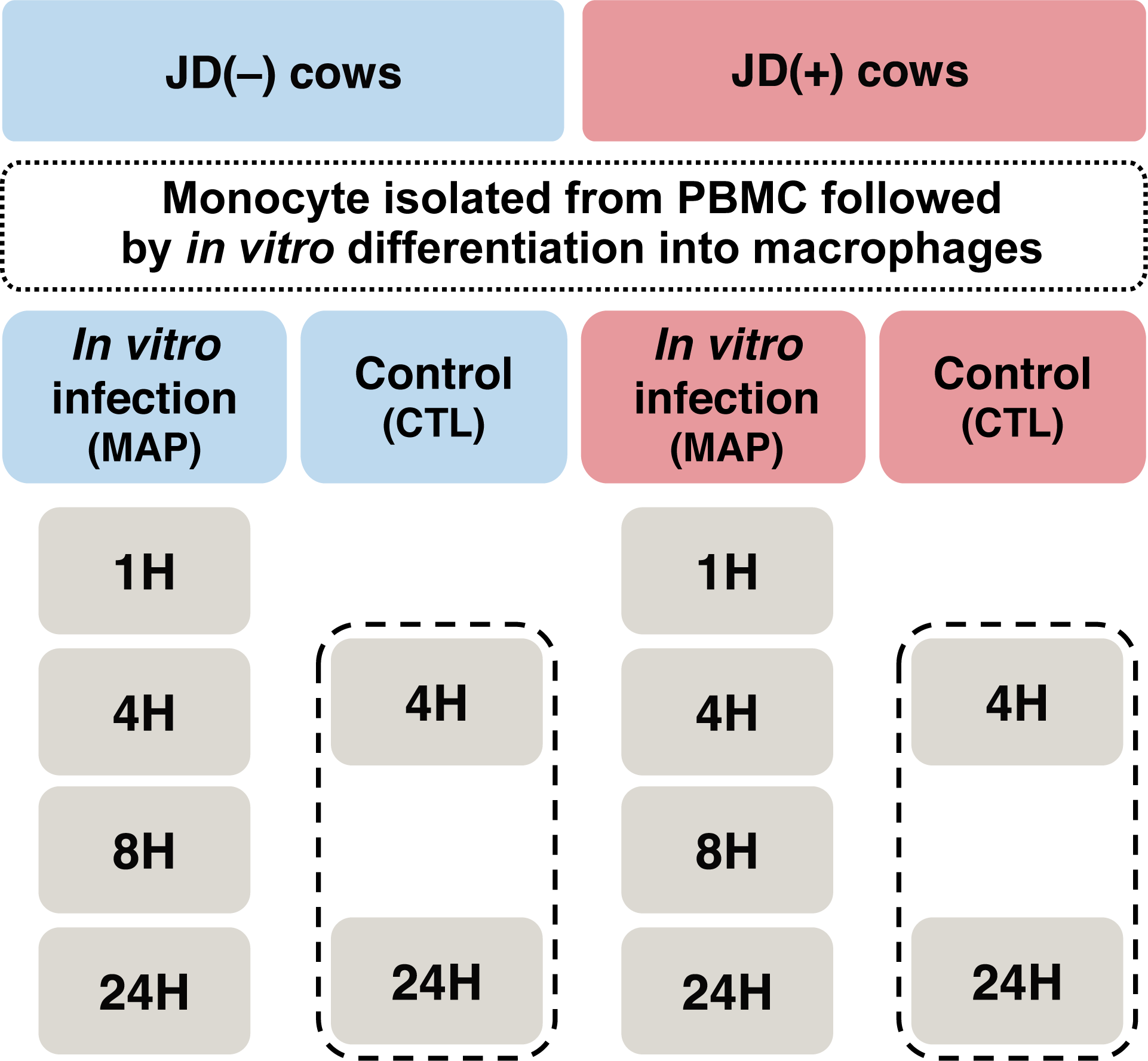

Supplement: Supplementary Figure 1 — Experimental design. Monocytes were isolated from Johne's disease negative [JD(–)] and positive [JD(+)] cows and cultured in vitro to support their differentiation into macrophages before the infection with live MAP at a MOI of 10:1. The 4 and 24 h controls (CTL), which are surrounded with a dotted line, were used as biological replicates for the RNA-seq and qPCR analyses. [file Image_1.TIF]

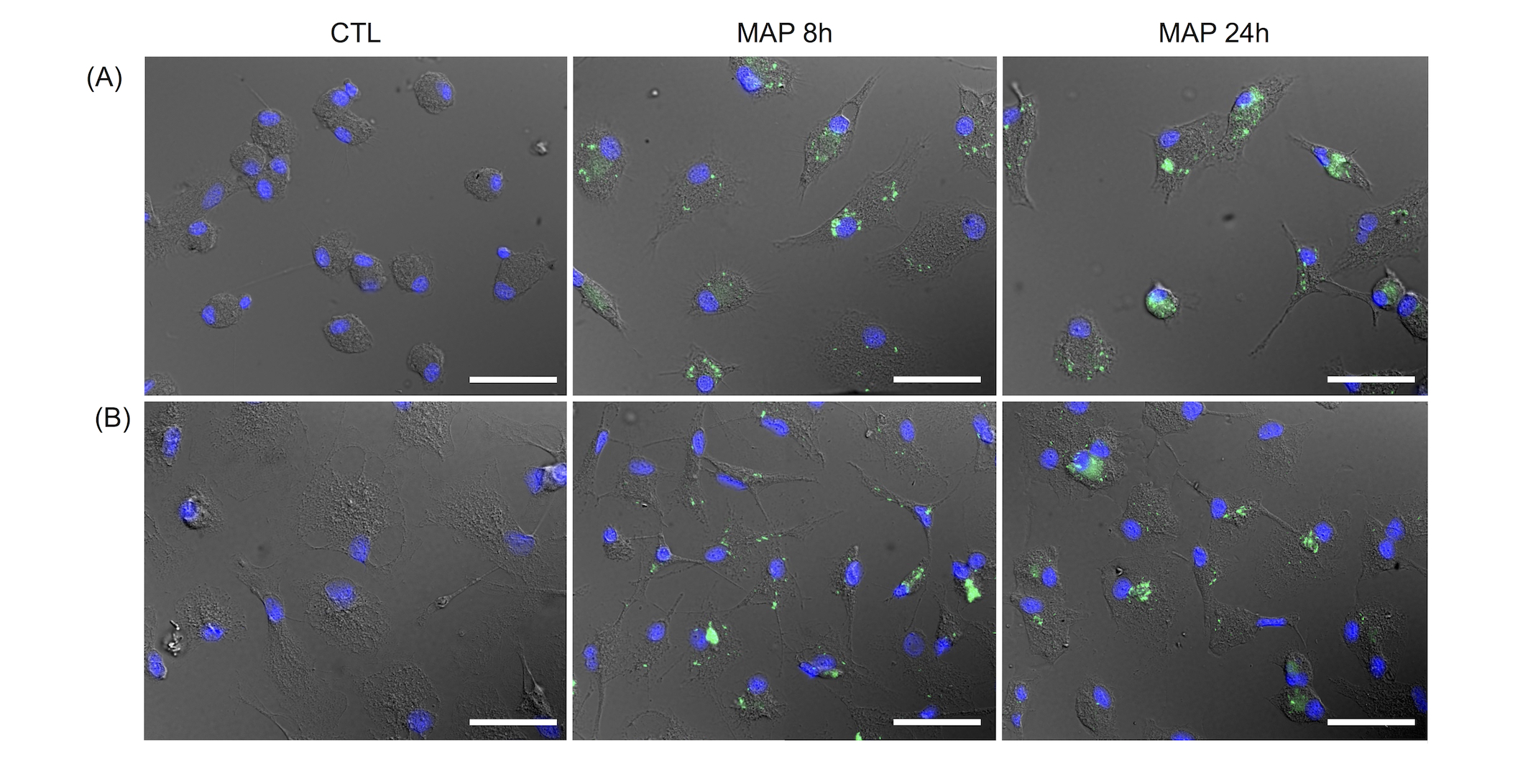

Supplement: Supplementary Figure 2 — Evaluation of MAP uptake by macrophages from Johne's disease negative (A) and Johne's disease positive (B) cows. The infection efficiency was evaluated in vitro using fluorescence microscopy after 8 and 24 h incubation with MAP. These images, which are representative of three independent experiments on three different cows, show control (CTL) uninfected cells and 8 or 24 hpi of MAP infected macrophages. The images were merged from an observation of stained mycobacteria shown in green (fluorescent Auramine-O staining), nucleic acid stained in blue (DAPI), and differential interference contrast at 630× magnification. Scale bar = 40 μm. [file Image_2.JPEG]

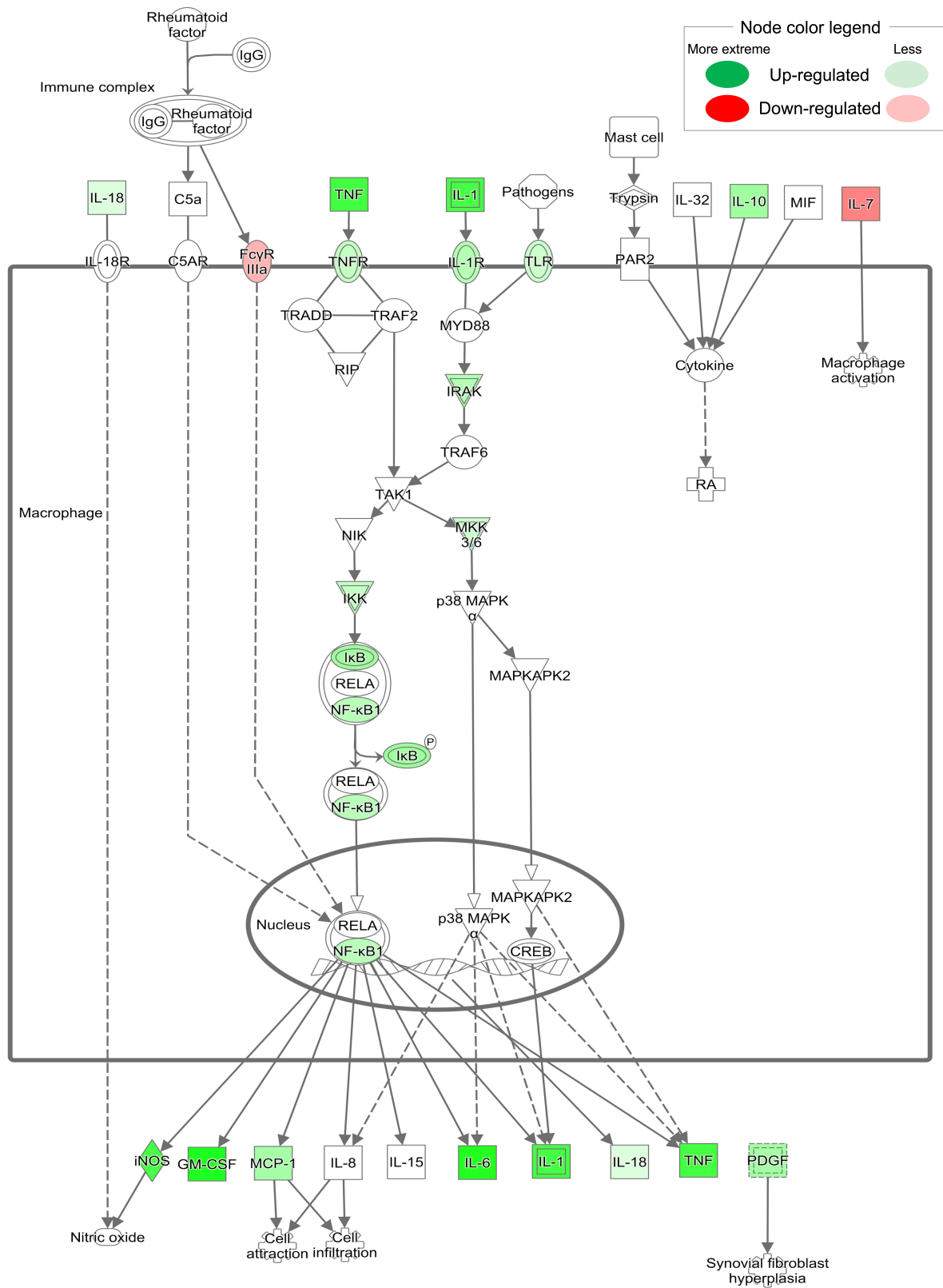

Supplement: Supplementary Figure 4 — Role of macrophages, fibroblasts and endothelial cells in rheumatoid arthritis pathway. This IPA pathway is the second most enriched by significant DE genes at 8hpi of JD(–) macrophages challenged with MAP. Shades of green represent the upregulated fold-change values of RNA-seq data and shades of red represent the downregulated values from MAP infected macrophages. [file Image_4.PDF]

(A)

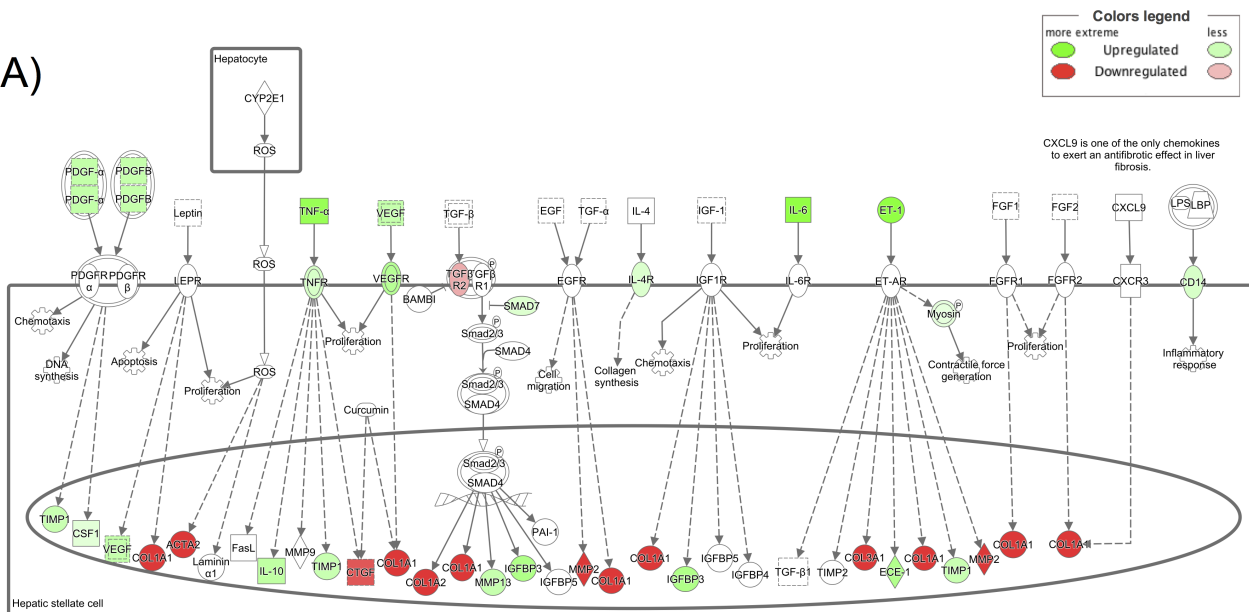

(B)

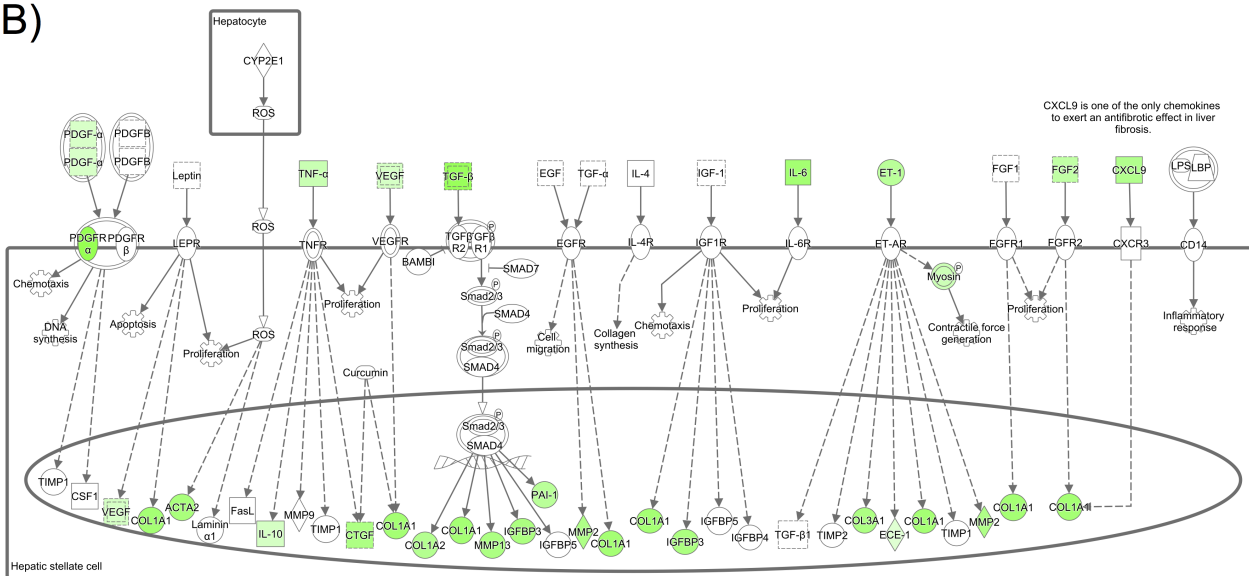

Supplement: Supplementary Figure 5 — Early signaling events in fat storing cells (also known as stellate cells). The IPA top-ranked “Hepatic fibrosis/hepatic stellate cell activation” pathway in primary MAP-infected macrophages: (A) at 8 hpi of JD(–) macrophages compared to CTL JD(–) and (B) for the comparison of CTL samples of JD(+) vs. JD(–) macrophages. Shades of green represent upregulated significant DE genes and shades of red represent the downregulated significant DE genes. [file Image_5.PDF]

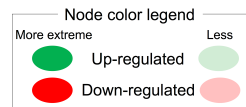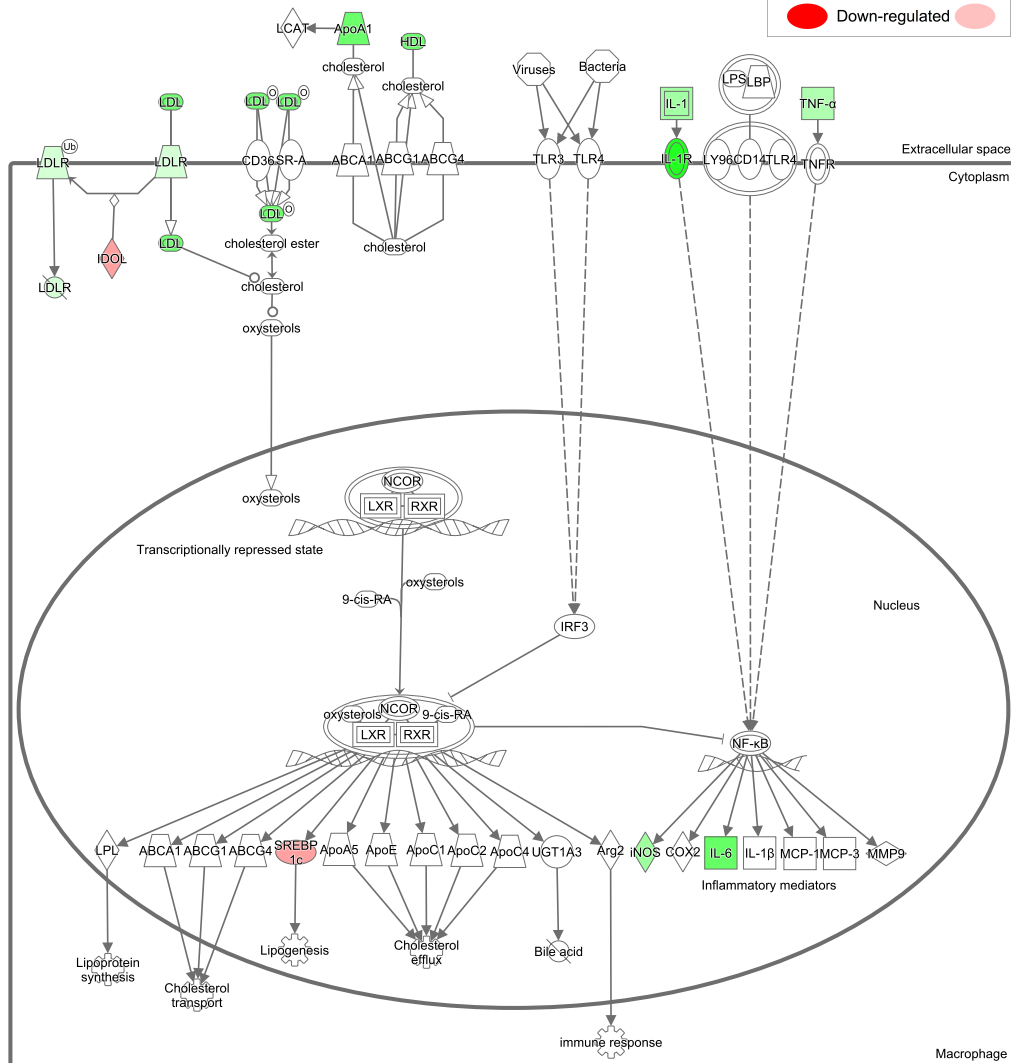

Supplement: Supplementary Figure 6 — “LXR/RXR activation” pathway significantly enriched by significant DE genes of JD(+) CTL macrophages. Shades of green represent upregulated genes compared to JD(–) macrophages, while shades of red represent downregulated genes as reported by the RNA-seq analysis. [file Image_6.PDF]
