# Supplementary material for: Transcriptome Profiling of Bovine Macrophages Infected by Mycobacterium avium spp. paratuberculosis Depicts Foam Cell and Innate Immune Tolerance Phenotypes
Source: Front Immunol. 2020 Jan 8;10:2874. doi: 10.3389/fimmu.2019.02874 (PMC6960179; doi:10.3389/fimmu.2019.02874)
Supplement: Supplementary Figure 3 — Lipid accumulation assay using Oil Red O staining for two additional cows for uninfected (control) or MAP-infected (5 days) experimental condition. The images were taken at (A) 200× magnification (scale bar = 200 μm) and at (B) a 400× magnification (scale bar = 100 μm). These are representative images from three experiments. [file Image_3.pdf]

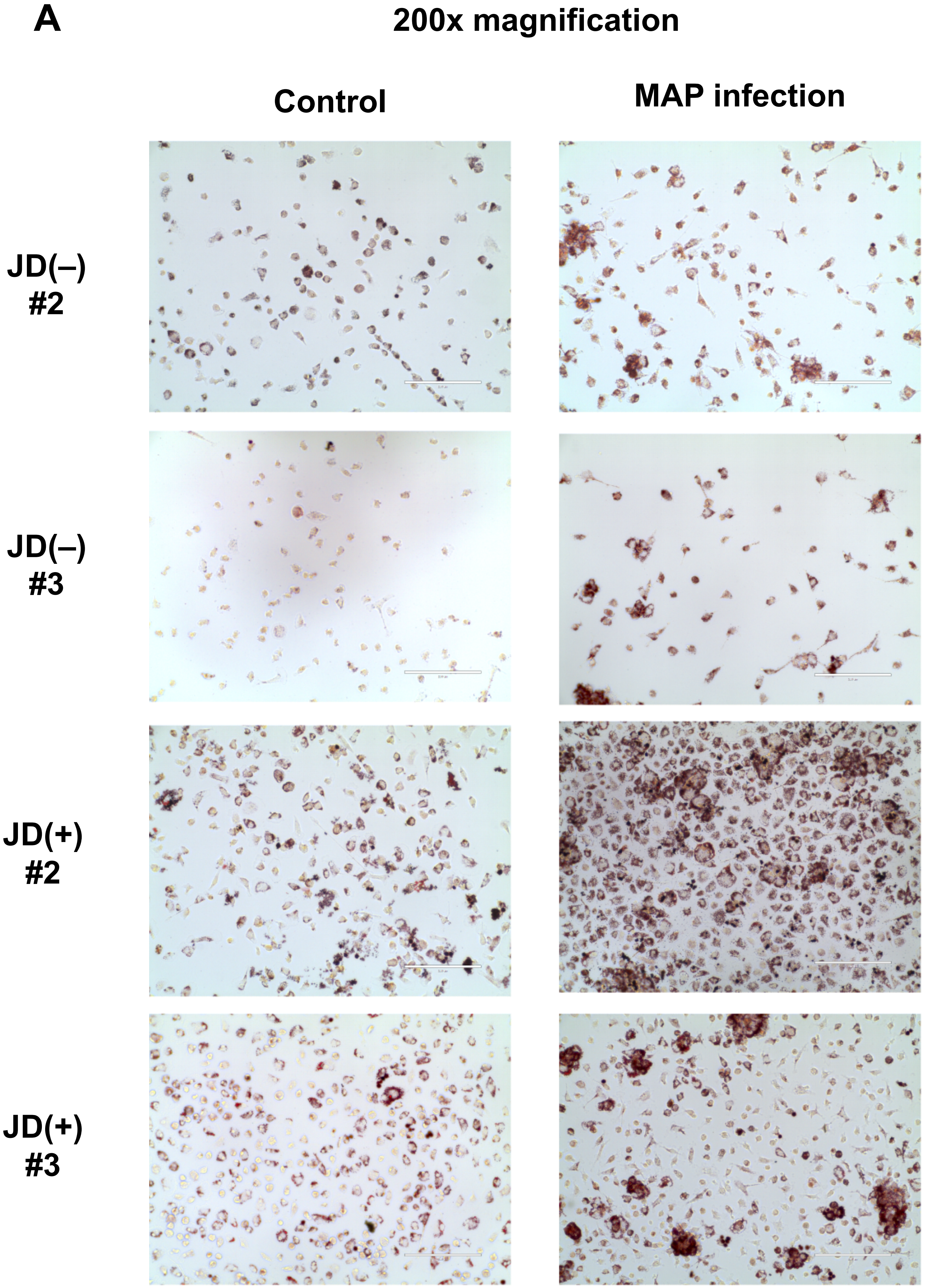
A
200x magnification
Control
MAP infection
JD(–) #2
JD(–) #3
JD(+) #2
JD(+) #3

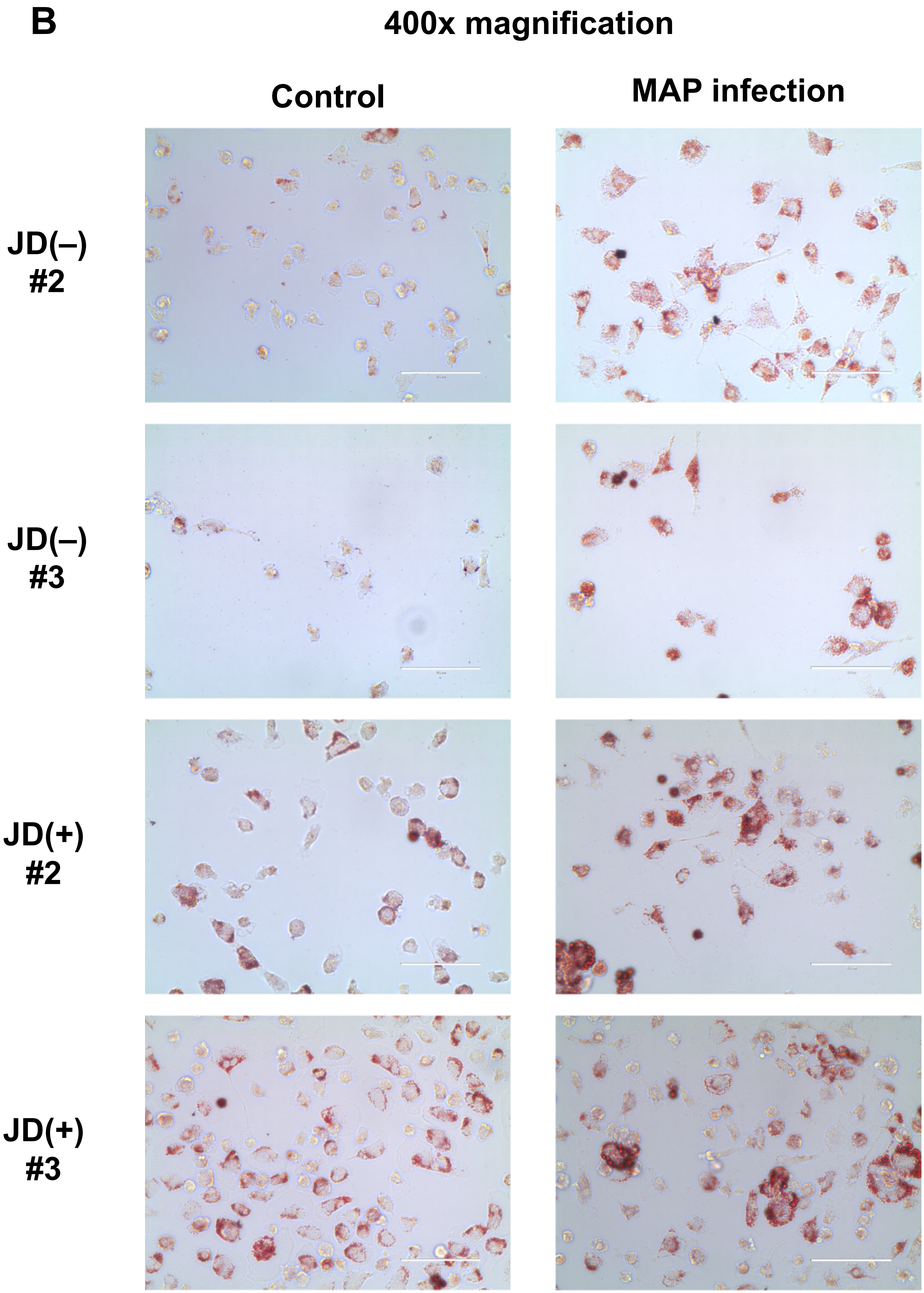
B
400x magnification
Control
MAP infection
JD(–) #2
JD(–) #3
JD(+) #2
JD(+) #3
